# Power analysis for RNA-Seq differential expression studies

**DOI:** 10.1186/s12859-017-1648-2

**Published:** 2017-05-03

**Authors:** Lianbo Yu, Soledad Fernandez, Guy Brock

**Affiliations:** Center for Biostatistics, Department of Biomedical Informatics, The Ohio State University, 1800 Cannon Dr., Columbus, 43210 OH USA

**Keywords:** RNA-Seq, Power, Wald test, Likelihood ratio test

## Abstract

**Background:**

Sample size calculation and power estimation are essential components of experimental designs in biomedical research. It is very challenging to estimate power for RNA-Seq differential expression under complex experimental designs. Moreover, the dependency among genes should be taken into account in order to obtain accurate results.

**Results:**

In this paper, we propose a simulation based procedure for power estimation using the negative binomial distribution and assuming a generalized linear model (at the gene level) that considers the dependence between gene expression level and its variance (dispersion) and also allows equal or unequal dispersion across conditions. We compared the performance of both Wald test and likelihood ratio test under different scenarios. The null distribution of the test statistics was simulated for the desired false positive control to avoid excess false positives with the usage of an asymptotic chi-square distribution. We applied this method to the TCGA breast cancer data set.

**Conclusions:**

We provide a framework for power estimation of RNA-Seq data. The proposed procedure is able to properly control the false positive error rate at the nominal level.

**Electronic supplementary material:**

The online version of this article (doi:10.1186/s12859-017-1648-2) contains supplementary material, which is available to authorized users.

## Background

Discovering differential expression has been the main focus of many biological experiments and many patient cohort studies for several decades. Since the invention of microarray chips twenty years ago, a huge amount of data has been generated by profiling thousands of genes in various cell lines, model organisms, and human samples. Recently, RNA-Seq technology became the replacement of array technology because of its ability to not only quantify the transcriptome but also detect gene isoforms, novel transcripts, and gene fusion [1–3]. Similar to microarray studies, sample size calculations and power estimation are still some of the key issues in designing RNA-Seq experiments, but face some new challenges given the nature of RNA-Seq data.

RNA-Seq studies generate count based data. Several earlier published papers used Poisson distribution to model the count data [4–6]. Due to the restraint of the mean equal to the variance under the Poisson distribution, the negative binomial (NB) distribution is a natural choice to provide a better fit for RNA-Seq data by allowing an over-dispersion parameter to capture extra variability over the mean. Thus, several specialized software packages have been developed to model RNA-Seq data based on the negative binominal distribution. Robinson et al. [7] developed the R package *edgeR*, which provides an exact test for two group comparisons initially and then was expanded to allow multifactor designs by a generalized linear model. Additionally, Love et al. [8] developed the R package *DESeq2* for differential expression analysis, which provides shrinkage estimators for both log fold change and dispersion by imposing a hierarchical model on them.

For testing differential expression with RNA-Seq experiments, several studies have attempted to provide sample size calculation and power estimation at a single gene level in the recent literature. Fang and Cui [6] introduced a simulation based power estimation approach using Wald test and likelihood ratio test (LRT). Li et al. [9] proposed an exact test method for calculating sample size at a single gene level or the marginal level, which is implemented in a web tool called *RNAseqPS* [10] and an R package called *RnaSeqSampleSize*. Other studies have been published for sample size calculation and power estimation at a data set level by evaluating the proportion of true discoveries. Shyr and Li [11] proposed a sample size calculation method using TCGA data. Ching et al. [12] simulated data from six public data sets and compared power in the paired and unpaired designs. The *PROPER* method by Wu et al. [13] is a prospective power assessment approach, which simulated data based on an actual RNA-Seq data set, assessed several empirical error rates and empirical power levels, and stratified them by mean expression and dispersion. However these methods require simulations of all genes based on pilot data or data with similar biological context, and the specification of effect sizes of all genes simultaneously is a big challenge.

The above-mentioned literature on RNA-Seq sample size calculation and power estimation employed common analysis approaches, such as edgeR or DESeq2, that assume the negative binomial distribution. However, all these NB-based approaches have resulted in an inflated type I error rate as reported in several papers [14–17]. Accurate sample size calculation and power estimation should rely on an appropritate control of false positive error rate. Thus the major contribution of our study is that we addressed this issue by using a simulation based empirical approach. This approach properly controls the false positive error rate at the desired level. The idea of using the simulation based approach was originally proposed for modeling brain lesion counts in a multiple slerosis clinical trial by Rettiganti and Nagaraja [18]. But in their study, a simulation based method, called an exact parametric test, was developed for determining the critical values for testing treatment effect. The authors showed that the chi-square test used for Wald, Score, and LRT fails to maintain the nominal significance level, especially for small sample size studies. To overcome this deficiency in sample size calculation and power estimation approaches and to accommodate designs with multiple groups or multiple factors, we provide a framework that can be implemented for power estimation. The proposed simulation based procedure at a single gene level or marginal level uses the exact parametric test for power estimation to ensure the false positive error rate is properly controlled at the nominal level. In addition, we extended this procedure to unequal dispersion parameter cases for RNA-Seq sample size calculation and power estimation, which has not been proposed before. Simulations were conducted for the proposed procedure under both scenarios.

## Methods

### Negative binomial model

A negative binomial random variable *X* with mean *μ* and dispersion *ϕ* is denoted as *N* *B*(*μ*,*ϕ*). It has variance *μ*+*μ* ^2^ *ϕ* and probability mass function as follows:

1 $${} P(X=x)=\frac{\Gamma(x+\phi^{-1})}{\Gamma(\phi^{-1})\Gamma(x+1)} \Big(\frac{1}{1+\mu\phi}\Big)^{\phi^{-1}} \Big(\frac{\mu}{\phi^{-1}+\mu}\Big)^{x},  $$

*x*=0,1,2,⋯ ;*μ*>0;*ϕ*>0.

### Dispersion as a function of mean expression

Love et al. [8] assume that the dispersion *ϕ* follows a log-Normal prior distribution with mean as a function of *μ*. The dispersion’s functional trend is modeled as

2 $$ \phi_{tr}(\mu) = \frac{a_{1}}{\mu} + a_{0}.   $$

To estimate this functional form, gene-wise dispersion estimators were regressed against the means of the normalized counts. This approach provides gene-wise shrinkage estimators of the dispersion parameter by assuming the mean-dispersion dependence for all genes and shows adequate power for detecting differential expression especially in small sample size experiments.

### Likelihood ratio test

Without loss of generality, we use *γ* to denote the fold ratio of a gene between two biological conditions. We are interested in testing the hypothesis *H*_0_:*γ*=1 vs. hypothesis *H*_1_:*γ*≠1. Let $x_{1},x_{2},\cdots,x_{n_{1}}\phantom {\dot {i}\!}$ and $y_{1},y_{2},\cdots,y_{n_{2}}\phantom {\dot {i}\!}$ represent the gene expression counts from each condition. The LRT statistic is given by

3 $$ L=-2log\bigg(\frac{sup_{\Theta_{0}}L(\mu,\gamma,\phi)}{sup_{\Theta}L(\mu,\gamma,\phi)}\bigg).  $$

According to Rettiganti and Nagaraja [18], the maximum likelihood estimate (MLE) of *μ* under *Θ*_0_ is

4 $$ \tilde{\mu}=\frac{n_{1}\bar{x}+n_{2}\bar{y}}{n_{1}+n_{2}}.  $$

While under *Θ*, the MLE of *μ* is $\bar {x}_{1}$, and the MLE of *γ* is $\bar {y}/\bar {x}$. Dispersion *ϕ* is estimated by numerically maximizing the likelihood.

### Wald test

A Wald test for testing log transformed *γ* with *H*_0_:*l* *o* *g*(*γ*)=0 vs. *H*_1_:*l* *o* *g*(*γ*)≠0 is given by

5 $$ W(log(\gamma))=\bigg(\frac{log(\hat{\gamma})}{\hat{\sigma}_{\gamma}/\hat{\gamma}}\bigg)^{2}.  $$

### False positive error rate control

With thousands of genes tested in an RNA-Seq study, multiple comparison adjustment is necessary. While the Bonferroni method for controlling the family-wise error rate (probability of one or more false rejections among all comparisons) is very conservative, a less conservative procedure, named the extended interpretation of the Bonferroni method, for controlling the mean number of false positives can be used for multiplicity adjustment [19]. In other words, the procedure controls the per family error rate (PFER) or per comparison error rate (PCER). It can be made as powerful as the Benjamimi-Hochberg FDR control procedure, and shows greater stability than the FDR. In our simulations, the nominal false positive error rate *α* will be the PCER for a single gene or at the marginal level.

### Empirical parametric test

Inferences on the Wald test and the LRT typically rely on the chi-square distribution by asymptotic theory for large sample sizes. But this may lead to liberal results for small sample sizes since asymptotic theory may not work as expected. To address this issue for small sample sizes, the simulation based test by Rettiganti and Nagaraja [18] is used to provide a proper false positive error rate control. In summary, the empirical null distribution of the test statistics (Wald or LRT) is obtained from simulated experimental data under the null hypothesis for a large number of iterations (e.g., 100,000). The 100(1−*α*)*t* *h* percentile from the null test statistics will be used as a significance cutoff for testing under the alternative hypothesis by comparing the test statistics with this percentile cutoff value.

### Power estimation procedure

1. Specify all input parameters: sample size per condition *n*; mean expression *μ*; fold ratio between conditions *γ*, nominal false positive error rate *α*, number of simulations *T*.
2. Estimate the mean-dispersion functional form using pilot data set or specify an assumed functional form.
3. Calculate dispersion *ϕ* using Eq. 2 with mean expression *μ*.
4. Simulate count data from *N* *B*(*μ*,*ϕ*)*T* times under both null and alternative hypotheses using the input parameters.
5. Fit NB model and obtain the test statistics (Wald or LRT) under the null hypothesis.
6. Calculate 100(1−*α*)*t* *h* percentile as the significance cutoff.
7. Fit NB model and obtain test statistics (Wald or LRT) under the alternative hypothesis.
8. Calculate power for the specified input parameters.

## Results

### Simulations

#### Parameter settings

**Fig. 1:**
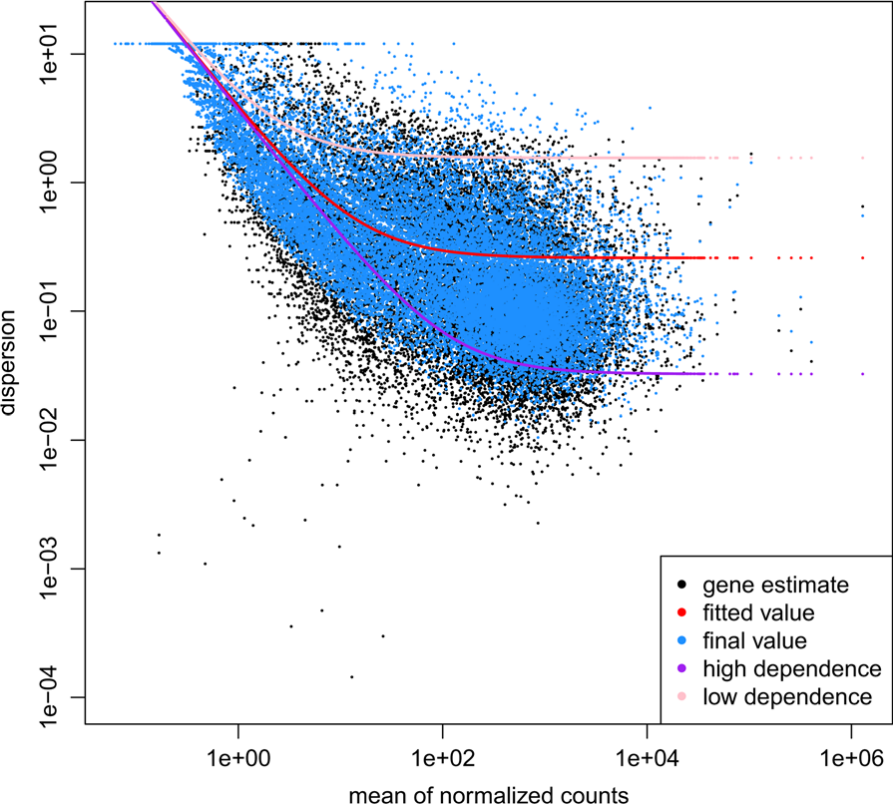
Mean-dispersion functional form for simulations. *DESeq2* method was applied on a pilot data of unpublished canine thyroid RNA-Seq data set for setting up simulation parameters. The plot shows the estimated mean-dispersion function form (*red dots*) relative to the mean of the normalized counts. *Black dots* represent per-gene estimates of the dispersion while *blue dots* represent moderated estimates calculated by *DESeq2*. The fitted functional form and a lower and higher dependency functional forms were used in the simulation studies

Count data were simulated from a negative binomial distribution under two experimental conditions (e.g. control vs. treatment) with equal dispersion parameters or unequal dispersion parameters (ratio of 1.5) between conditions. The parameters needed to calculate power of a single gene or marginal level are sample size per condition *n*, mean expression *μ* of control group, treatment-to-control fold ratio *γ*, and nominal false positive error rate *α*. Simulation settings were $n=5, 10, 20, 30, 40; \mu =4, 20, 100, 500; \gamma =\frac {1}{3},\frac {1}{2},\frac {1}{1.5},1,1.5,2,3; \alpha =0.01, 0.005, 0.001, 0.0005$. The dispersion parameter *ϕ* was calculated for each *μ* from the mean-dispersion functional form *ϕ*=0.26+3.65/*μ* estimated from an unpublished canine thyroid RNA-Seq data set (Fig. 1). To explore the effect of the mean-dispersion functional form, a low-dependency (*ϕ*=1.56+3.65/*μ*) and a high-dependency (*ϕ*=0.032+3.65/*μ*) were also considered in the simulations as shown in Fig. 1. At each setting, 100,000 simulations were run under the null hypothesis and 10,000 simulations were run under the alternative hypothesis. The critical value was estimated by the empirical 100(1−*α*)*t* *h* percentile from the null Wald and LRT statistics.

#### Equal dispersion

**Fig. 2:**
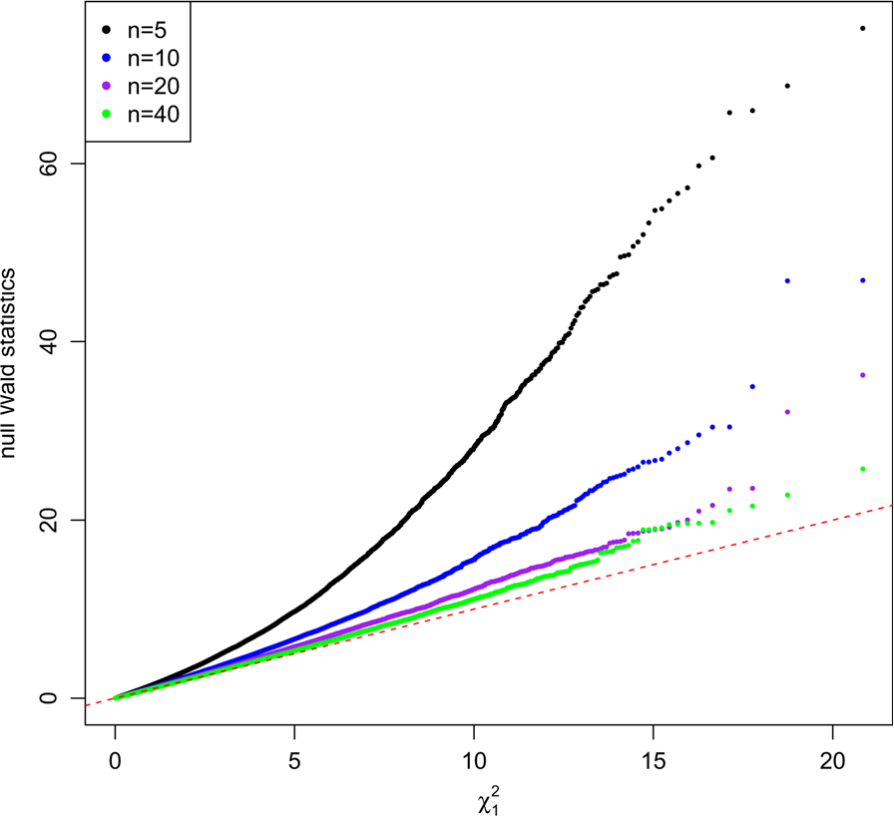
QQ plot of null Wald statistics with equal dispersion parameters. Data were simulated 100,000 times with *μ*=20 under the null hypothesis. Sample sizes were set at *n*=5,10,20,and 40. Wald test was used for testing mean difference between two conditions. The discrepancy of the null Wald statistics from chi-square distribution with 1 degree of freedom gets smaller when sample size increases

**Fig. 3:**
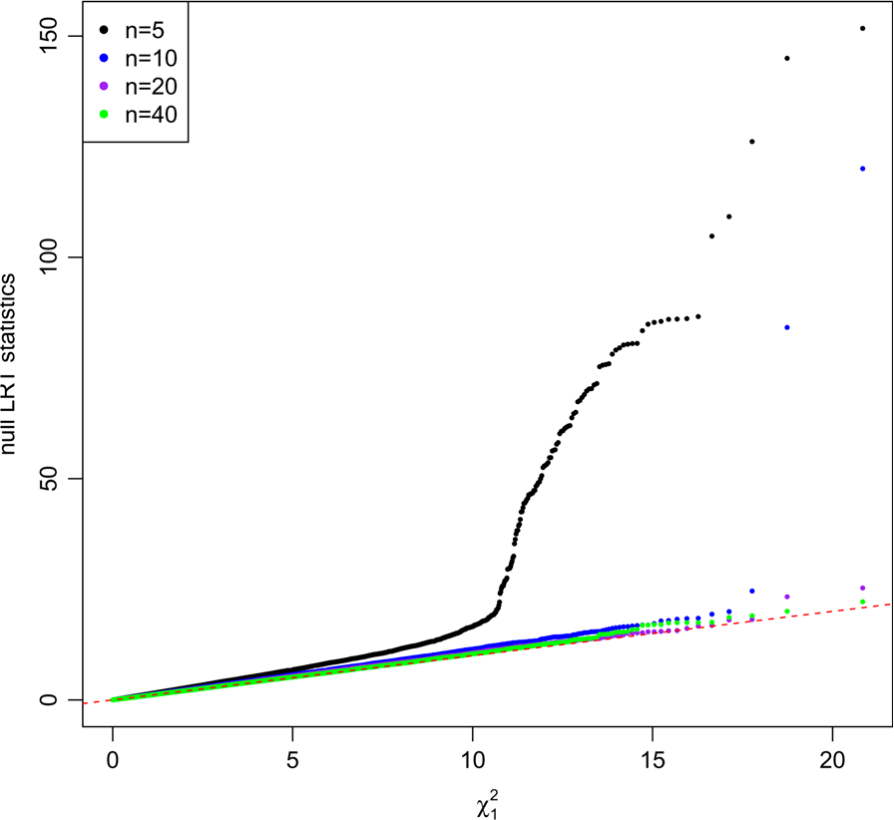
QQ plot of null LRT statistics with equal dispersion parameters. Data were simulated 100,000 times with *μ*=20 under the null hypothesis. Sample sizes were set at *n*=5,10,20,and 40. The LRT was used for testing mean difference between two conditions. The discrepancy of the null LRT statistics from chi-square distribution with 1 degree of freedom gets smaller when sample size increases

**Fig. 4:**
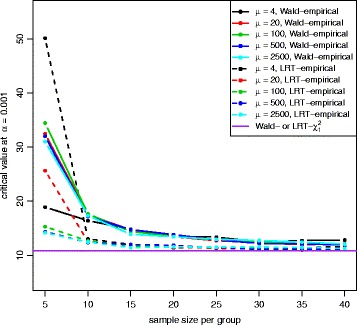
Critival values plot for both Wald test and LRT with equal dispersion parameters. Critical values were calculated at the nominal false positive error rate of 0.001 from empirical percentile of null statisitics at 5 different mean expression levels for both Wald test (*solid line*) and LRT (*dashed line*), and for the chi-square distribution with 1 degree of freedom (*purple line*). Both Wald test and LRT with the empirical distribution have larger critical values than both Wald test and LRT with the chi-square distribution, and the Wald test has much larger values than the LRT with the empirical distribution

**Fig. 5:**
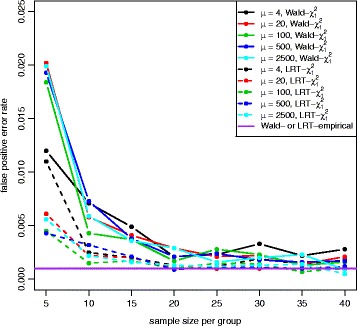
False positive error rate plot for both Wald test and LRT with equal dispersion parameters. False positive error rate was calculated for both Wald test (*solid line*) and LRT (*dashed line*) following a chi-square distribution with 1 degree of freedom at 5 different mean expression levels. The nominal false positive error rate for both Wald and LRT with the empirical distribution is shown in *purple line* (*α*=0.001). Both tests with the chi-square distribution have the inflated false positive error rates

Figures 2 and 3 show the QQ plots for the Wald statistics and the LRT statistics under the null hypothesis at sample size *n*=5,10,20,40 with mean expression *μ*=20. When sample size increases, the distribution of either Wald or LRT statistics converges toward the chi-square distribution with 1 degree of freedom with a faster convergence for the LRT. The discrepancy is quite large when the sample size is small. Figure 4 shows the critical values using the empirical parametric test and the chi-square distribution at 8 different sample sizes and 5 different mean expression levels for both Wald test and LRT. The critical values get smaller with larger sample sizes. The empirical parametric approach for both Wald test and LRT has much higher critical values than the chi-square distribution at smaller sample sizes, and the differences decrease when the sample size gets larger. The Wald test has larger critical values than the LRT in general. At each sample size and each mean expression level, the false positive error rate is controlled at the nominal level by the empirical parametric test. However, the estimated false positive error rate of either Wald test or LRT (Fig. 5) following the asymptotic chi-square distribution with 1 degree of freedom is much larger than the nominal false positive error rate, especially for small sample sizes.

**Fig. 6:**
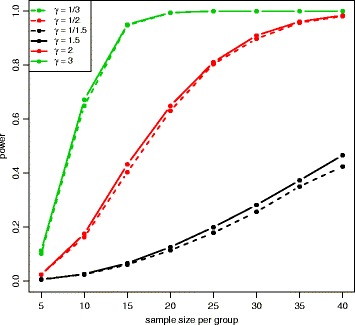
Power plot at *μ*=100 for the Wald test with equal dispersion parameters. Power was calculated at 8 different sample sizes and 6 different fold changes under the alternative hypothesis with *μ*=100 and *α*=0.001. Power is higher for larger sample sizes and higher absolute fold changes

**Fig. 7:**
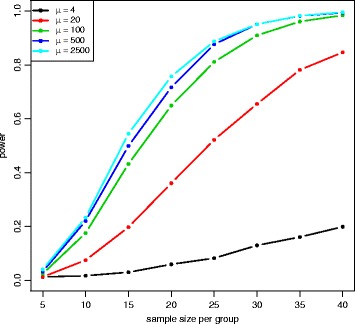
Power plot at *γ*=2 for the Wald test with equal dispersion parameters. Power was calculated at 8 different sample sizes and 5 different expression levels with *γ*=2 under the alternative hypothesis and *α*=0.001. Power is higher for larger sample sizes and higher expression levels

Figure 6 and Additional file 1: Figure S1 show power at 8 different sample sizes and 6 different fold changes with mean expression *μ*=100 for the Wald test and the LRT at *α*=0.001. In both plots, power increases with larger sample sizes and larger absolute fold changes. Figure 7 and Additional file 1: Figure S2 show power at 8 different sample sizes and 5 different mean expression level with fold change *γ*=2 under the alternative hypothesis and *α*=0.001. In both plots, power increases with larger sample sizes and larger mean expression levels. The Wald test and the LRT have similar power at different parameter values. Compared to the results for the medium-dependency functional form, both low-dependency and high-dependency functional forms have similar critical values and false positive error rates, but power estimation is lower(higher) for low(high)-dependency. (See Additional file 1: Figures S3–S8 for results using *α*=0.01 and Additional file 1: Figures S9–S20 for results with low(high)-dependency functional form).

#### Unequal dispersion

**Fig. 8:**
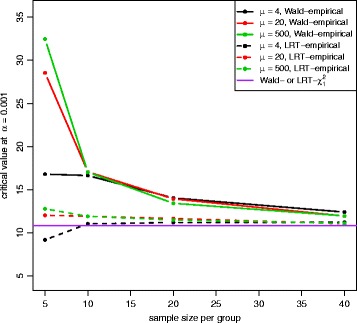
Critival values plot for both Wald test and LRT with unequal dispersion parameters. Critical values were calculated at the nominal false positive error control level of 0.001 from empirical percentile of null statisitics at 3 different mean expression levels for both Wald test (*solid line*) and LRT (*dashed line*), and for a chi-square distribution with 1 degree of freedom (*purple line*). Both Wald test and LRT have larger critical values than the chi-square distribution, and the Wald test has much larger values than the LRT

**Fig. 9:**
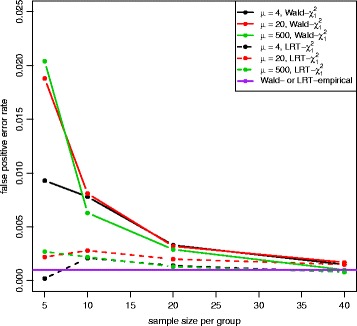
False positive error rate plot for both Wald test and LRT with unequal dispersion parameters. False positive error rate was calculated for both Wald test (*solid line*) and LRT (*dashed line*) following a chi-square distribution with 1 degree of freedom at 3 different mean expression levels. The nominal false positive error rate control level for the empirical parametric test is shown in *purple line* (*α*=0.001). Both tests have inflated false positive error rates

**Fig. 10:**
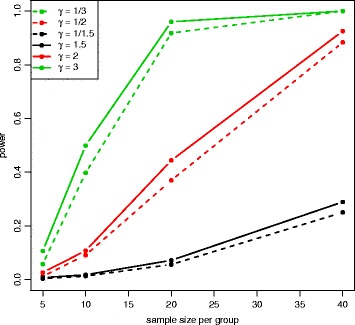
Power plot at *μ*=20 for the Wald test with unequal dispersion parameters. Power was calculated at 4 different sample sizes and 6 different fold changes under the alternative hypothesis with *μ*=20 and *α*=0.001. Power is higher for larger sample sizes and higher absolute fold changes

**Fig. 11:**
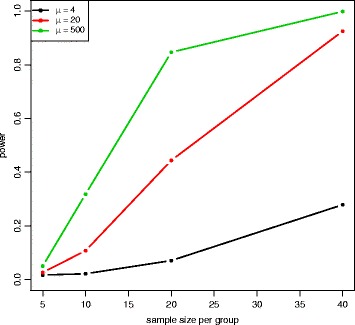
Power plot at *γ*=2 for the Wald test with unequal dispersion parameters. Power was calculated at 4 different sample sizes and 3 different expression levels with *γ*=2 under the alternative hypothesis and *α*=0.001. Power is higher for larger sample sizes and higher expression levels

The QQ plot of the Wald test under the unequal dispersion setting (Additional file 1: Figure S21) is similar to the QQ plot of the equal dispersion setting, but the LRT (Additional file 1: Figure S22) has minor differences between QQ plots of different sample sizes. Figure 8 shows the critical values of the empirical parametric distribution and the chi-square distribution at 4 different sample sizes and 3 different mean expression levels for both Wald test and LRT. Similar to the equal dispersion setting, the empirical parametric test of the Wald test has much higher critical values than the chi-square distribution at small sample sizes, and the differences get smaller when sample size gets larger. However, the LRT has slightly higher critical values than the chi-square distribution. For false positive error rate (Fig. 9), the Wald test with the chi-square distribution has much higher values than the nominal level, while the LRT with the chi-square distribution has slightly higher values. Similar to the equal dispersion setting, the power of the Wald test (Figs. 10 and 11) and the LRT (Additional file 1: Figures S23 and S24) at *α*=0.001 is increased with larger sample sizes, larger mean expression levels, and larger absolute fold changes. (See Additional file 1: Figures S25–S30 for results using *α*=0.01 and Additional file 1: Figures S31–S42 for results with low(high)-dependency functional form).

### Applications

#### TCGA data set

**Fig. 12:**
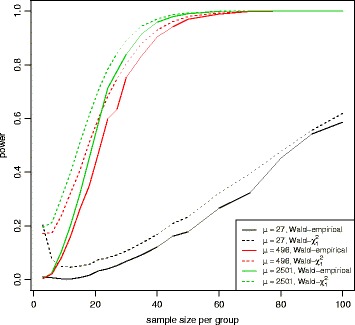
Power plot for the Wald test with equal dispersion parameters for TCGA breast cancer data set. Power was calculated for the Wald test with the empirical distribution (*solid line*) or with a chi-square distribution with 1 degree of freedom (*dotted line*) at 3 different mean expression levels, 19 different sample sizes (range 3-100), and a fold change of 2 under the alternative hypothesis with *α*=0.001

**Fig. 13:**
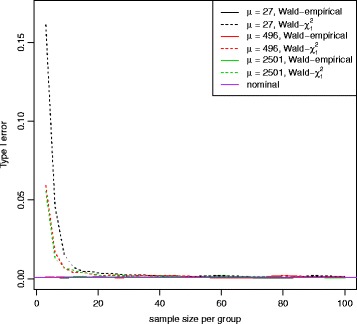
False positive error rate plot for the Wald test with equal dispersion parameters for TCGA breast cancer data set. False positive error rate was calculated for the Wald test with the empirical distribution (*solid line*) or with a chi-square distribution with 1 degree of freedom (*dotted line*) at 3 different mean expression levels and 19 different sample sizes (range 3-100). The nominal false positive error rate for the Wald test with the empirical distribution is shown in *purple line* (*α*=0.001). Wald test with the chi-square distribution has the inflated false positive error rates

To study and demonstrate the proposed power estimation procedure in a real data application, we used the TCGA breast cancer data set as a pilot data for designing a new study for detecting differential expression. The TCGA breast cancer data set, acquired in Sep. 2015 from cBioPortal for Cancer Genomics, contains 1003 tumor samples with clinical information and 17866 gene features with non-zero counts. We chose the comparison between two tumor stage categories I-II (746 samples) vs. III-IV (238 samples) when fitting the *DESeq2* package for estimating the mean-dispersion functional form (Additional file 1: Figure S43). The estimated mean expression levels were 27, 496, 2501 at the 10*t* *h*, 50*t* *h*, 90*t* *h* percentiles for all gene features, respectively. Figure 12 shows the power as a function of sample sizes (range 3-100) at these mean expression percentiles for a 2-fold difference between two patient subgroups. Wald test for the proposed empirical distribution and for the chi-square distribution were used at *α*=0.001. Figure 13 shows the false positive error rate at *α*=0.001. Even though the Wald test for the chi-square distribution has a little higher power at smaller sample sizes, this is mainly due to the failure to properly control false positive rate. To design a new study with 80% power, we will need *n*=33 samples per group to detect a 2-fold difference for genes at the mean expression level of 496. The computation time for this power estimation is about 8 hours on a standard windows laptop with Intel Core i7-6820HQ CPU at 2.70GHz and 32GB RAM.

## Discussion

Many published methods on identifying differentially expressed genes are based on the negative binomial distribution, and the inference mainly relies on asymptotic theory which is biased for small sample sizes. Several studies by Leng et al. [14], Lund et al. [15], Reeb and Steibel [16], and Rocke et al. [17] have reported the excess false positives by using these methods for differential expression detection with RNA-Seq data. The main reason is that the use of the significance cutoff from biased asymptotic distribution leads to the inflated false positive error rate especially for small sample sizes. Our simulation results confirm the great downward bias in the significance cutoff values when an asymptotic chi-square distribution is applied for both Wald test and LRT. Using the empirical parametric test for estimating the critical values, we are able to control the false positive error rate at the desired nominal level for both tests (Additional file 1: Figures S44–S45).

In all current published methods on differential expression detection and power estimation, the dispersion parameter is assumed equal across conditions. Under this assumption, the power will be misestimated if dispersion values are very different across conditions. Therefore in the simulations we allowed the dispersion parameter to be equal or unequal across conditions to achieve accurate power. When the dispersion parameter is assumed equal, the exact test method by Li et al. [9] can be used for power estimation. However this exact test method only works for two group comparisons and it can not be adapted to allow for unequal dispersion across conditions.

The proposed work not only can be applied to multiple groups and multiple factor designs through generalized linear models, it can also be extended to the data set level. In this case, the null distribution of the test statistics could be simulated for each gene or for a group of genes with similar expression profile for a proper control of the false positive error rate.

## Conclusions

With the emergence of RNA-Seq technology in recent years, RNA-Seq experiments have been widely used as an alternative to microarrays in biomedical research. Due to different data types, data analysis and power estimation are also different. New methods on sample size calculations and power estimation using the negative binomial distribution have already been proposed for this new technology. To overcome some of the limitations in current methods, we provide a framework for power estimation of RNA-Seq experiments by proposing a simulation based procedure, which provides a proper false positive control and can be applied in generalized linear model settings.

## Additional file

Additional file 1 This file provides all supplementary figures referenced in results section. **Figure S1.** Power plot at *μ*=100 for the LRT with equal dispersion parameters. **Figure S2.** Power plot at *γ*=2 for the LRT with equal dispersion parameters. **Figure S3.** Critival values plot for both Wald test and LRT with equal dispersion parameters at *α*=0.01. **Figure S4.** False positive error rate plot for both Wald test and LRT with equal dispersion parameters at *α*=0.01. **Figure S5.** Power plot at *μ*=100 for the Wald test with equal dispersion parameters at *α*=0.01. **Figure S6.** Power plot at *μ*=100 for the LRT with equal dispersion parameters at *α*=0.01. **Figure S7.** Power plot at *γ*=2 for the Wald test with equal dispersion parameters at *α*=0.01. **Figure S8.** Power plot at *γ*=2 for the LRT with equal dispersion parameters at *α*=0.01. **Figure S9.** Critival values plot for both Wald test and LRT with equal dispersion parameters assuming the high dependency functional form. **Figure S10.** False positive error rate plot for both Wald test and LRT with equal dispersion parameters assuming the high dependency functional form. **Figure S11.** Power plot at *μ*=100 for the Wald test with equal dispersion parameters assuming the high dependency functional form. **Figure S12.** Power plot at *μ*=100 for the LRT with equal dispersion parameters assuming the high dependency functional form. **Figure S13.** Power plot at *γ*=2 for the Wald test with equal dispersion parameters assuming the high dependency functional form. **Figure S14.** Power plot at *γ*=2 for the LRT with equal dispersion parameters assuming the high dependency functional form. **Figure S15.** Critival values plot for both Wald test and LRT with equal dispersion parameters assuming the low dependency functional form. **Figure S16.** False positive error rate plot for both Wald test and LRT with equal dispersion parameters assuming the low dependency functional form. **Figure S17.** Power plot at *μ*=100 for the Wald test with equal dispersion parameters assuming the low dependency functional form. **Figure S18.** Power plot at *μ*=100 for the LRT with equal dispersion parameters assuming the low dependency functional form. **Figure S19.** Power plot at *γ*=2 for the Wald test with equal dispersion parameters assuming the low dependency functional form. **Figure S20.** Power plot at *γ*=2 for the LRT with equal dispersion parameters assuming the low dependency functional form. **Figure S21.** QQ plot of null Wald statistics with unequal dispersion parameters. **Figure S22.** QQ plot of null LRT statistics with unequal dispersion parameters. **Figure S23.** Power plot at *μ*=20 for the LRT with unequal dispersion parameters. **Figure S24.** Power plot at *γ*=2 for the LRT with unequal dispersion parameters. **Figure S25.** Critival values plot for both Wald test and LRT with unequal dispersion parameters at *α*=0.01. **Figure S26.** False positive error rate plot for both Wald test and LRT with unequal dispersion parameters at *α*=0.01. **Figure S27.** Power plot at *μ*=20 for the Wald test with unequal dispersion parameters at *α*=0.01. **Figure S28.** Power plot at *μ*=20 for the LRT with unequal dispersion parameters at *α*=0.01. **Figure S29.** Power plot at *γ*=2 for the Wald test with unequal dispersion parameters at *α*=0.01. **Figure S30.** Power plot at *γ*=2 for the LRT with unequal dispersion parameters at *α*=0.01. **Figure S31.** Critival values plot for both Wald test and LRT with unequal dispersion parameters assuming the high dependency functional form. **Figure S32.** False positive error rate plot for both Wald test and LRT with unequal dispersion parameters assuming the high dependency functional form. **Figure S33.** Power plot at *μ*=20 for the Wald test with unequal dispersion parameters assuming the high dependency functional form. **Figure S34.** Power plot at *μ*=20 for the LRT with unequal dispersion parameters assuming the high dependency functional form. **Figure S35.** Power plot at *γ*=2 for the Wald test with unequal dispersion parameters assuming the high dependency functional form. **Figure S36.** Power plot at *γ*=2 for the LRT with unequal dispersion parameters assuming the high dependency functional form. **Figure S37.** Critival values plot for both Wald test and LRT with unequal dispersion parameters assuming the low dependency functional form. **Figure S38:** False positive error rate plot for both Wald test and LRT with unequal dispersion parameters assuming the low dependency functional form. **Figure S39.** Power plot at *μ*=20 for the Wald test with unequal dispersion parameters assuming the low dependency functional form. **Figure S40.** Power plot at *μ*=20 for the LRT with unequal dispersion parameters assuming the low dependency functional form. **Figure S41.** Power plot at *γ*=2 for the Wald test with unequal dispersion parameters assuming the low dependency functional form. **Figure S42.** Power plot at *γ*=2 for the LRT with unequal dispersion parameters assuming the low dependency functional form. **Figure S43.** Mean-dispersion functional form of TCGA breast cancer data set. **Figure S44.** False positive error rate plot for both Wald test and LRT with equal dispersion parameters. **Figure S45.** False positive error rate plot for both Wald test and LRT with unequal dispersion parameters. (PDF 355 kb)

## Abbreviations

LRT: Likelihood ratio test
MLE: Maximum likelihood estimation
NB: Negative binomial
PCER: Per comparison error rate
PFER: Per family error rate
TCGA: The cancer genome atlas
