# Supplementary material for: Power analysis for RNA-Seq differential expression studies
Source: BMC Bioinformatics. 2017 May 3;18:234. doi: 10.1186/s12859-017-1648-2 (PMC5415728; doi:10.1186/s12859-017-1648-2)
Supplement: Additional file 1 — This file provides all supplementary figures referenced in results section. Figure S1. Power plot at μ=100 for the LRT with equal dispersion parameters. Figure S2. Power plot at γ=2 for the LRT with equal dispersion parameters. Figure S3. Critival values plot for both Wald test and LRT with equal dispersion parameters at α=0.01. Figure S4. False positive error rate plot for both Wald test and LRT with equal dispersion parameters at α=0.01. Figure S5. Power plot at μ=100 for the Wald test with equal dispersion parameters at α=0.01. Figure S6. Power plot at μ=100 for the LRT with equal dispersion parameters at α=0.01. Figure S7. Power plot at γ=2 for the Wald test with equal dispersion parameters at α=0.01. Figure S8. Power plot at γ=2 for the LRT with equal dispersion parameters at α=0.01. Figure S9. Critival values plot for both Wald test and LRT with equal dispersion parameters assuming the high dependency functional form. Figure S10. False positive error rate plot for both Wald test and LRT with equal dispersion parameters assuming the high dependency functional form. Figure S11. Power plot at μ=100 for the Wald test with equal dispersion parameters assuming the high dependency functional form. Figure S12. Power plot at μ=100 for the LRT with equal dispersion parameters assuming the high dependency functional form. Figure S13. Power plot at γ=2 for the Wald test with equal dispersion parameters assuming the high dependency functional form. Figure S14. Power plot at γ=2 for the LRT with equal dispersion parameters assuming the high dependency functional form. Figure S15. Critival values plot for both Wald test and LRT with equal dispersion parameters assuming the low dependency functional form. Figure S16. False positive error rate plot for both Wald test and LRT with equal dispersion parameters assuming the low dependency functional form. Figure S17. Power plot at μ=100 for the Wald test with equal dispersion parameters assuming the low dependency functional form. [file 12859_2017_1648_MOESM1_ESM.pdf]

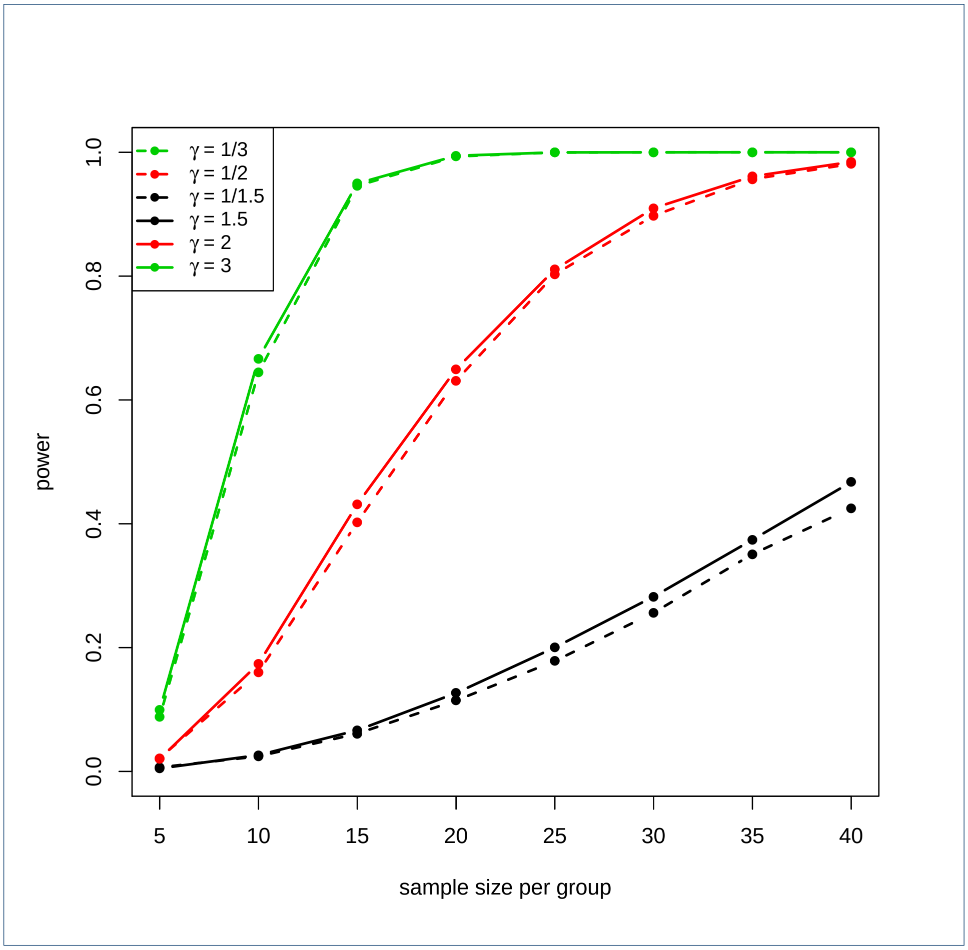

Figure S1**: Power plot at $\mu = 100$ for the LRT with equal dispersion parameters.** Power was calculated at 8 different sample sizes and 6 different fold changes under the alternative hypothesis with $\mu = 100$ and $\alpha = 0.001$. Power is higher for larger sample sizes and higher absolute fold changes.

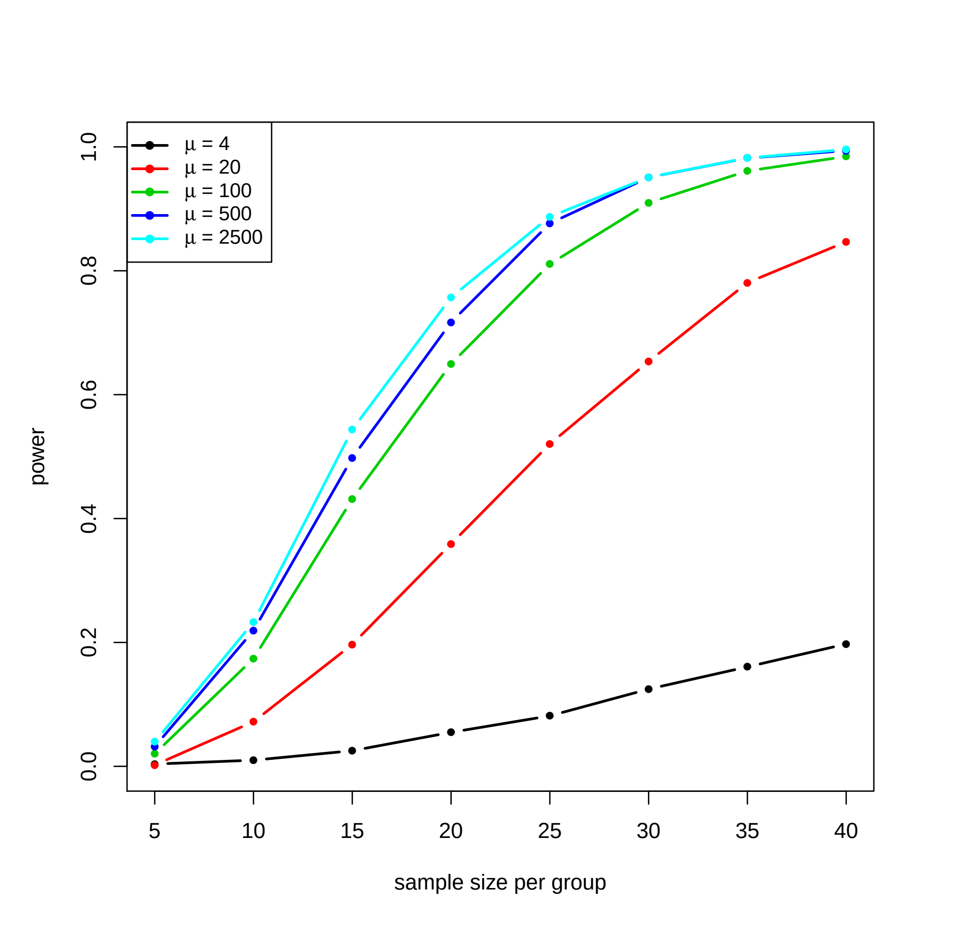

Figure S2: Power plot at $\gamma = 2$ for the LRT with equal dispersion parameters. Power was calculated at 8 different sample sizes and 5 different expression levels with $\gamma = 2$ under the alternative hypothesis and $\alpha = 0.001$. Power is higher for larger sample sizes and higher expression levels.

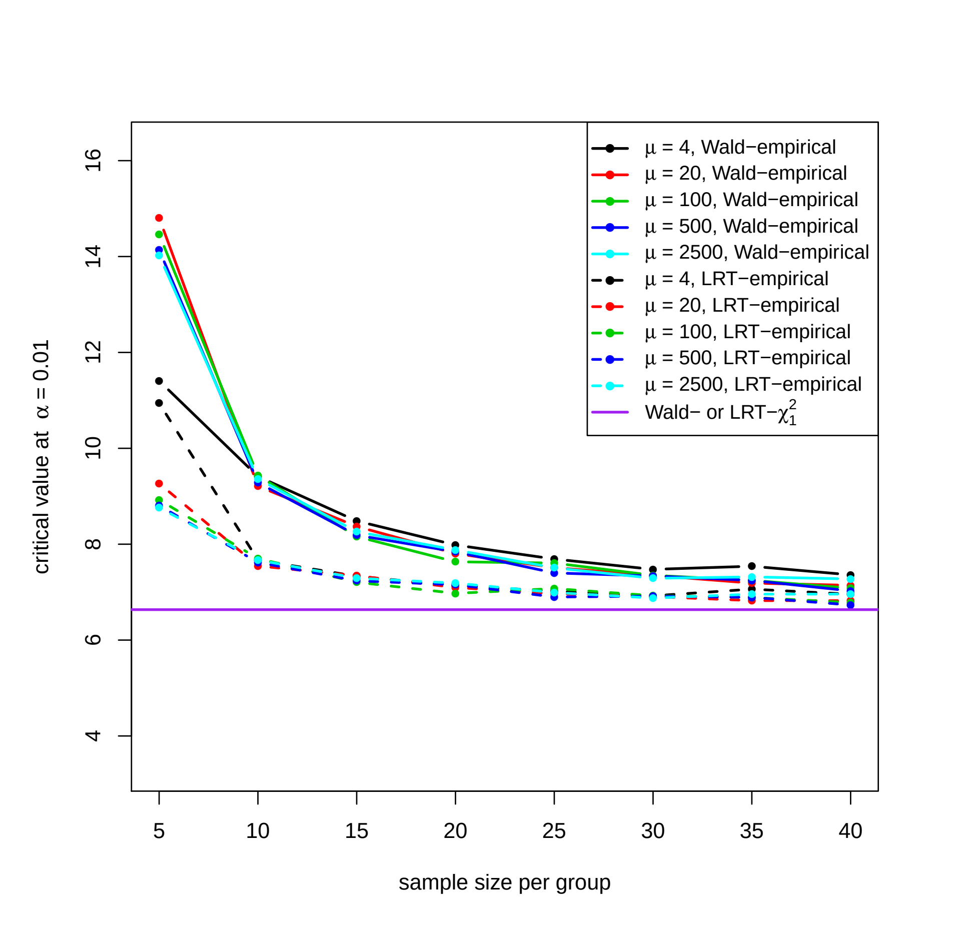

Figure S3: Critival values plot for both Wald test and LRT with equal dispersion parameters at $\alpha = 0.01$. Critical values were calculated at the nominal false positive error rate of 0.01 from empirical percentile of null statisitics at 5 different mean expression levels for both Wald test (solid line) and LRT (dashed line), and for the chi-square distribution with 1 degree of freedom (purple line). Both Wald test and LRT with the empirical distribution have larger critical values than both Wald test and LRT with the chi-square distribution.

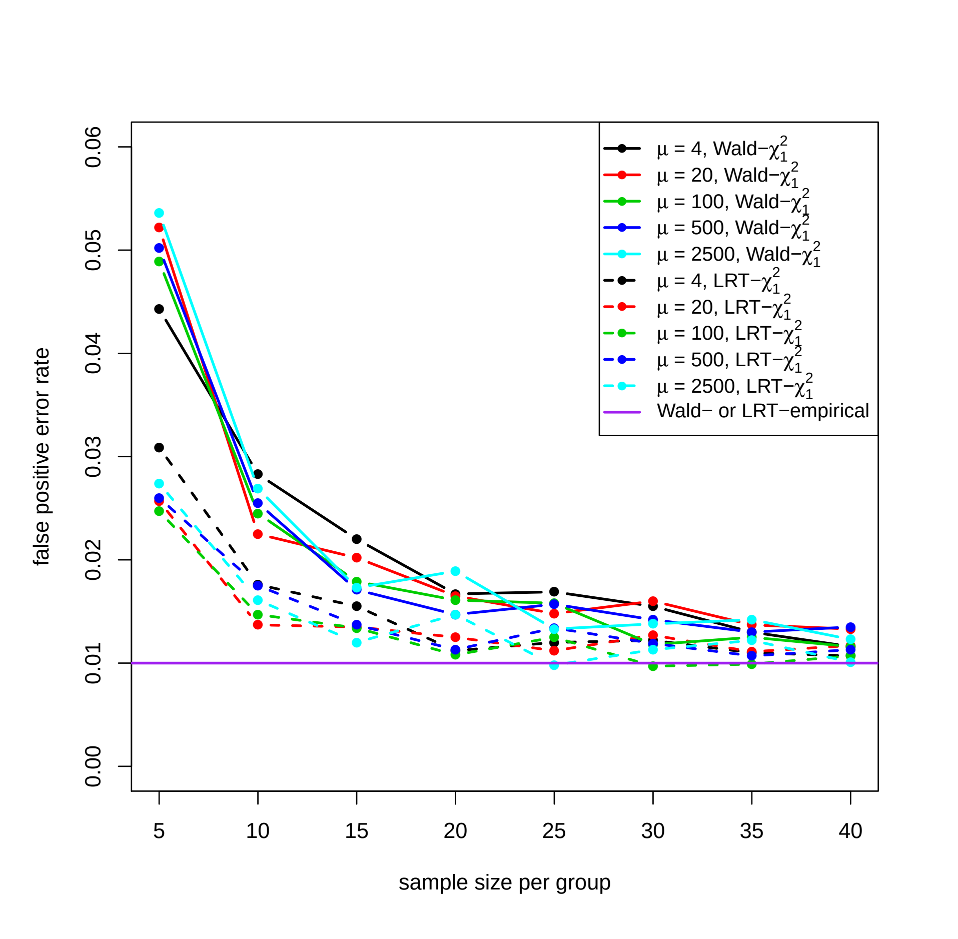

Figure S4: False positive error rate plot for both Wald test and LRT with equal dispersion parameters at $\alpha = 0.01$. False positive error rate was calculated for both Wald test (solid line) and LRT (dashed line) following a chi-square distribution with 1 degree of freedom at 5 different mean expression levels. The nominal false positive error rate for both Wald and LRT with the empirical distribution is shown in purple line ($\alpha = 0.01$). Both tests with the chi-square distribution have the inflated false positive error rates.

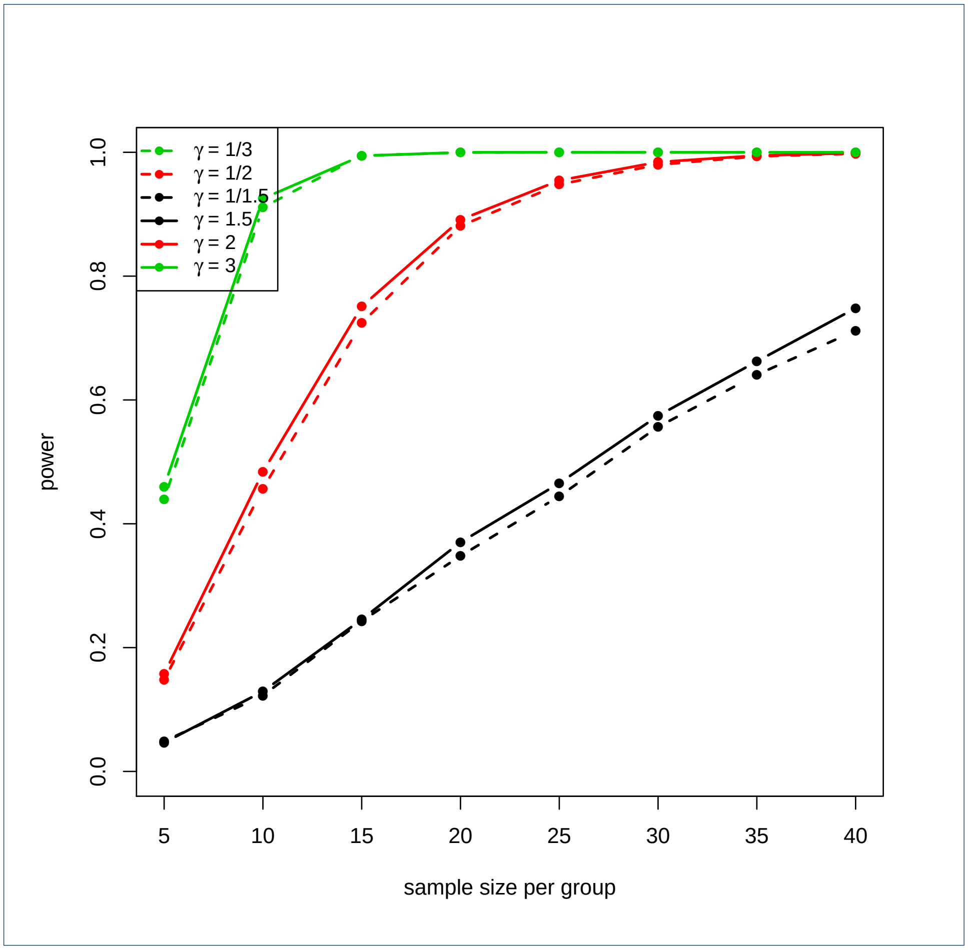

Figure S5: Power plot at $\mu = 100$ for the Wald test with equal dispersion parameters at $\alpha = 0.01$. Power was calculated at 8 different sample sizes and 6 different fold changes under the alternative hypothesis with $\mu = 100$ and $\alpha = 0.01$. Power is higher for larger sample sizes and higher absolute fold changes.

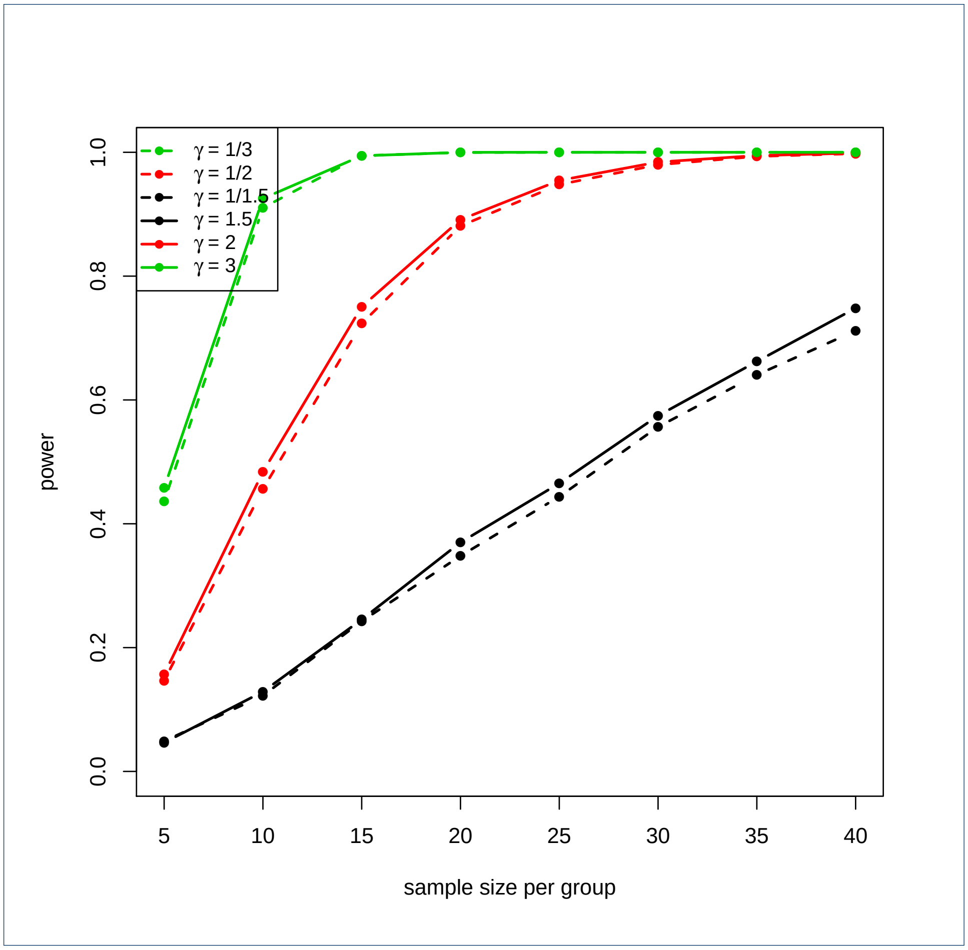

Figure S6: Power plot at $\mu = 100$ for the LRT with equal dispersion parameters at $\alpha = 0.01$. Power was calculated at 8 different sample sizes and 6 different fold changes under the alternative hypothesis with $\mu = 100$ and $\alpha = 0.01$. Power is higher for larger sample sizes and higher absolute fold changes.

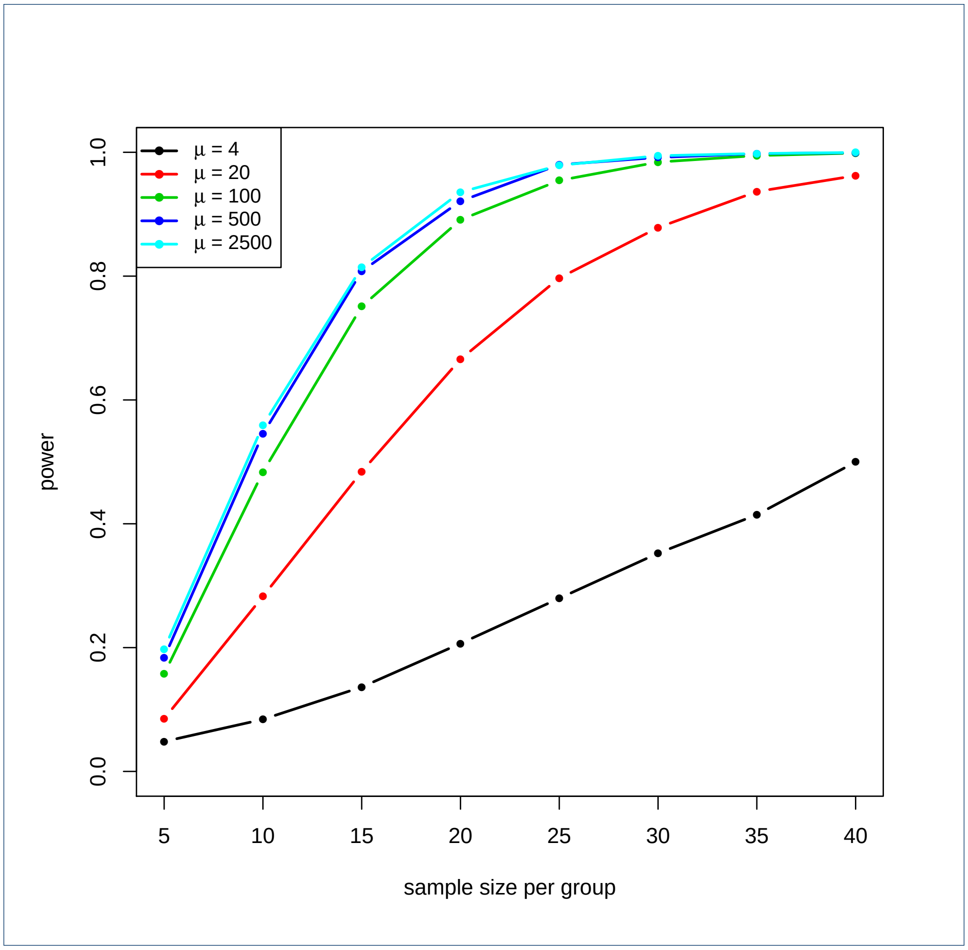

Figure S7: Power plot at $\gamma = 2$ for the Wald test with equal dispersion parameters at $\alpha = 0.01$. Power was calculated at 8 different sample sizes and 5 different expression levels with $\gamma = 2$ under the alternative hypothesis and $\alpha = 0.01$. Power is higher for larger sample sizes and higher expression levels.

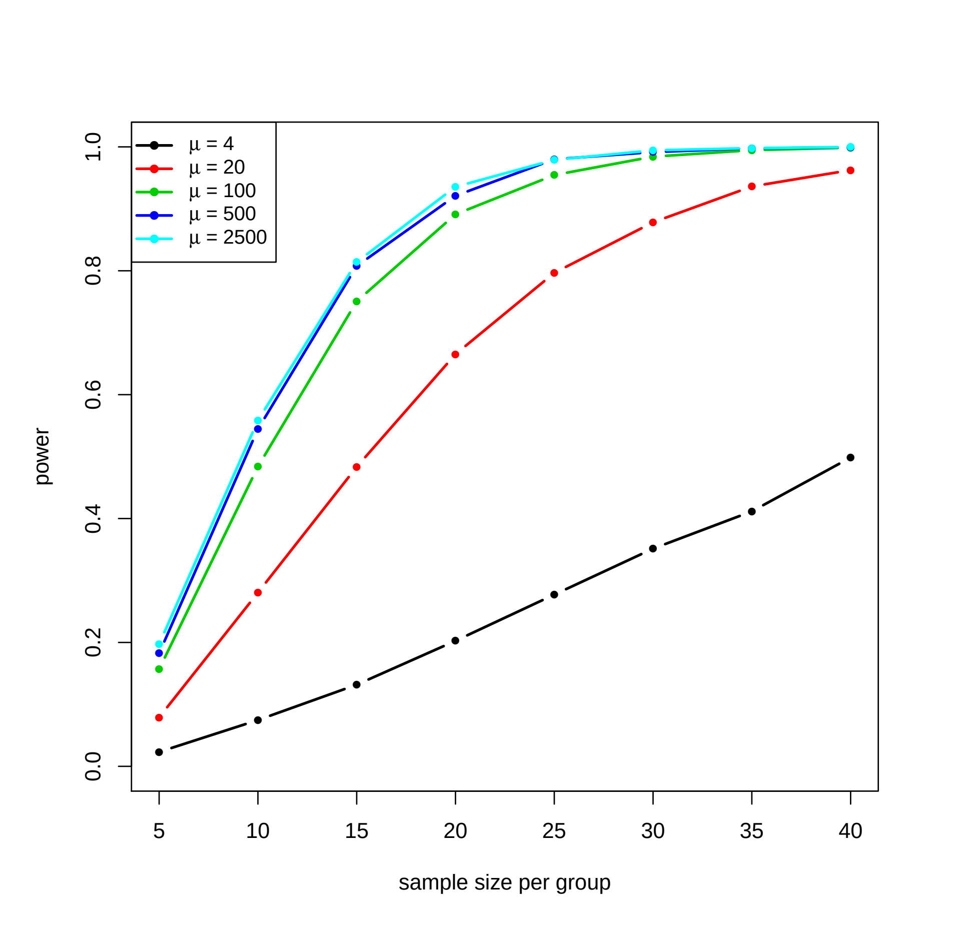

Figure S8: Power plot at $\gamma = 2$ for the LRT with equal dispersion parameters at $\alpha = 0.01$. Power was calculated at 8 different sample sizes and 5 different expression levels with $\gamma = 2$ under the alternative hypothesis and $\alpha = 0.01$. Power is higher for larger sample sizes and higher expression levels.

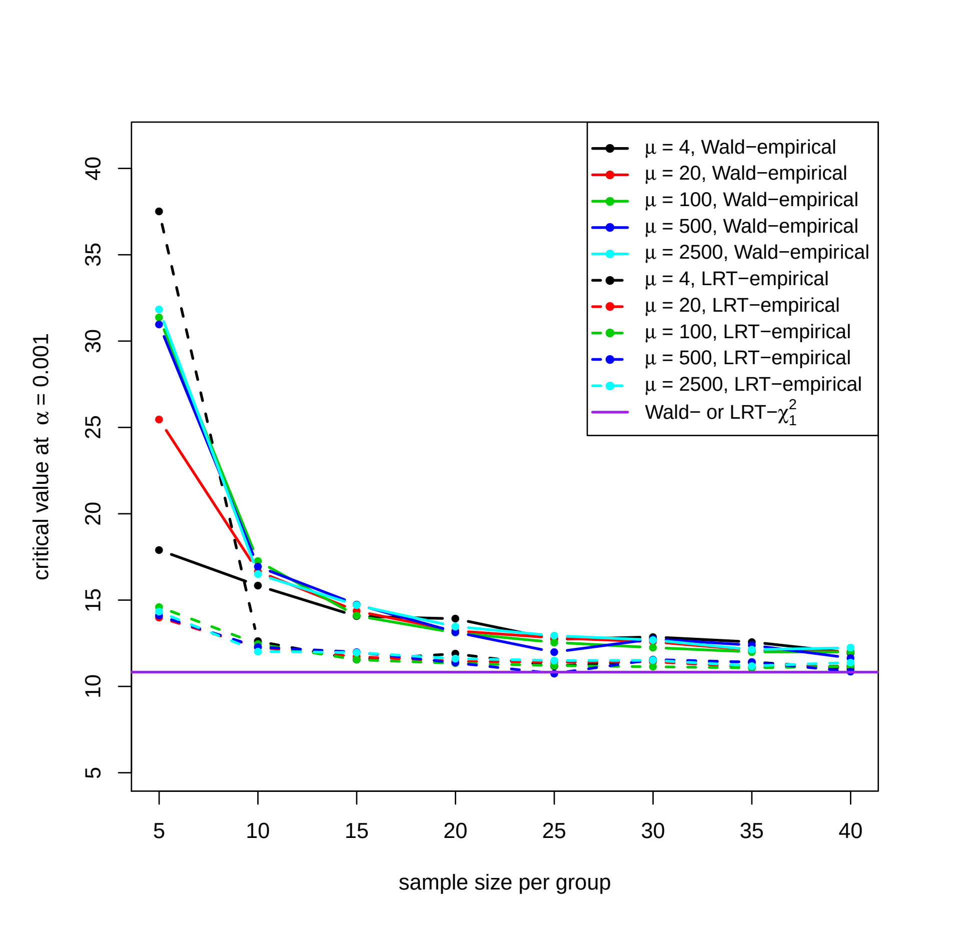

Figure S9: Critival values plot for both Wald test and LRT with equal dispersion parameters assuming the high dependency functional form. Critical values were calculated at the nominal false positive error rate of 0.001 from empirical percentile of null statisitics at 5 different mean expression levels for both Wald test (solid line) and LRT (dashed line), and for the chi-square distribution with 1 degree of freedom (purple line). Both Wald test and LRT with the empirical distribution have larger critical values than both Wald test and LRT with the chi-square distribution.

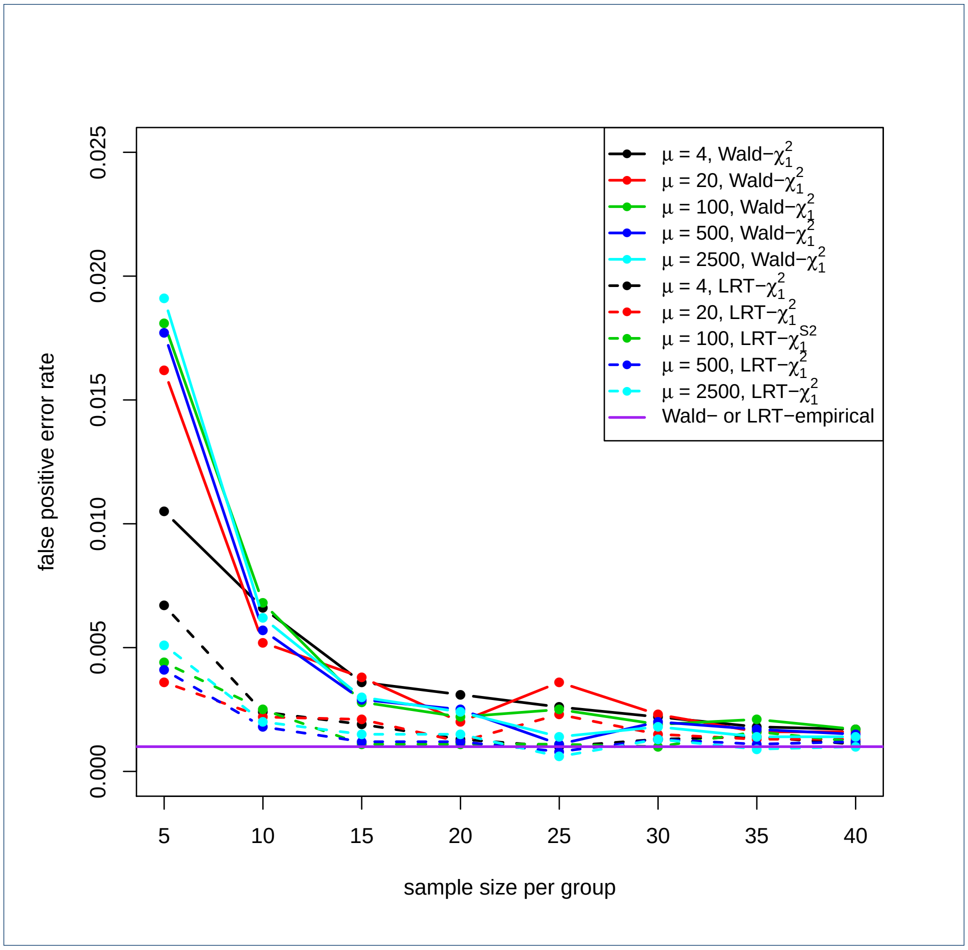

Figure S10: False positive error rate plot for both Wald test and LRT with equal dispersion parameters assuming the high dependency functional form. False positive error rate was calculated for both Wald test (solid line) and LRT (dashed line) following a chi-square distribution with 1 degree of freedom at 5 different mean expression levels. The nominal false positive error rate for both Wald and LRT with the empirical distribution is shown in purple line ($\alpha = 0.001$). Both tests with the chi-square distribution have the inflated false positive error rates.

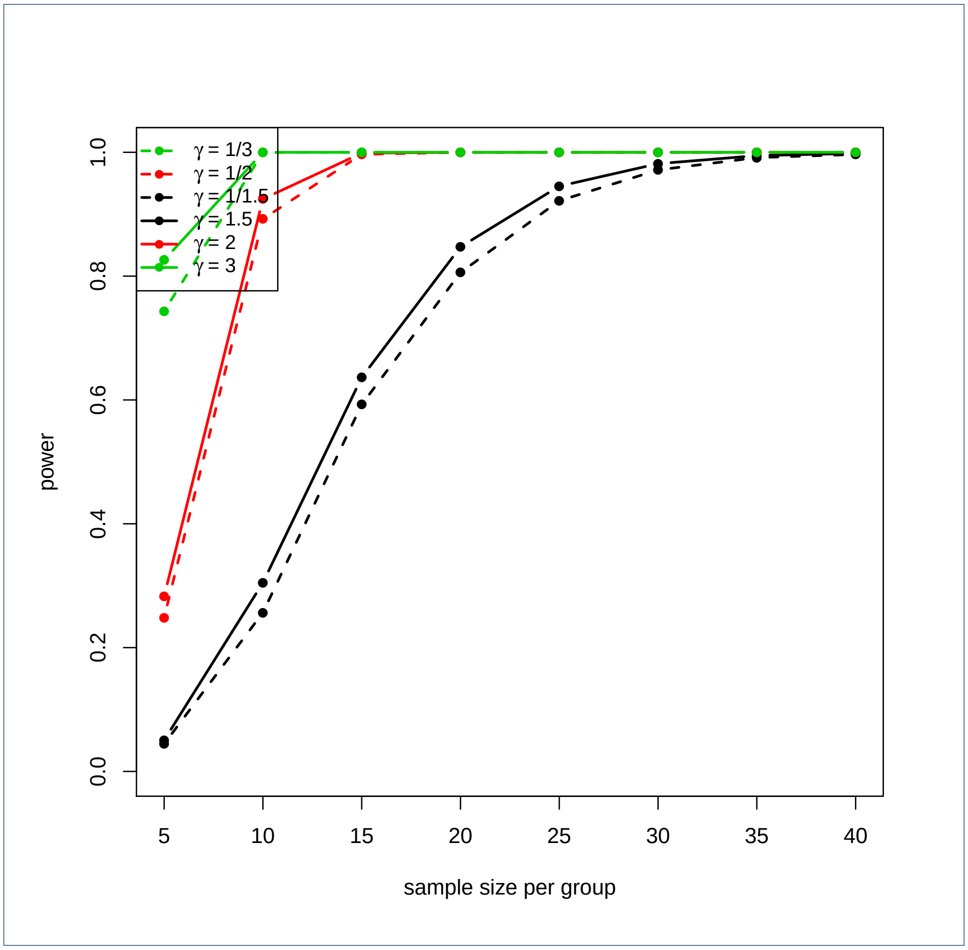

Figure S11: Power plot at $\mu = 100$ for the Wald test with equal dispersion parameters assuming the high dependency functional form. Power was calculated at 8 different sample sizes and 6 different fold changes under the alternative hypothesis with $\mu = 100$ and $\alpha = 0.001$. Power is higher for larger sample sizes and higher absolute fold changes.

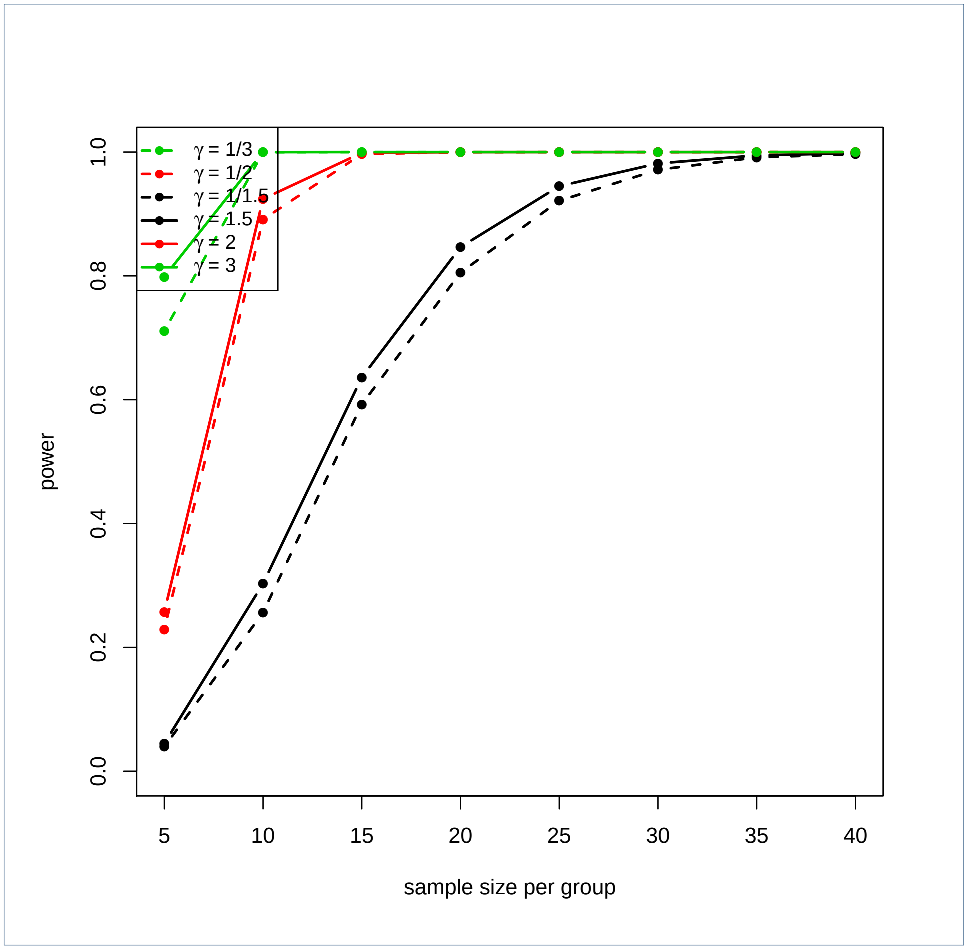

Figure S12: Power plot at $\mu = 100$ for the LRT with equal dispersion parameters assuming the high dependency functional form. Power was calculated at 8 different sample sizes and 6 different fold changes under the alternative hypothesis with $\mu = 100$ and $\alpha = 0.001$. Power is higher for larger sample sizes and higher absolute fold changes.

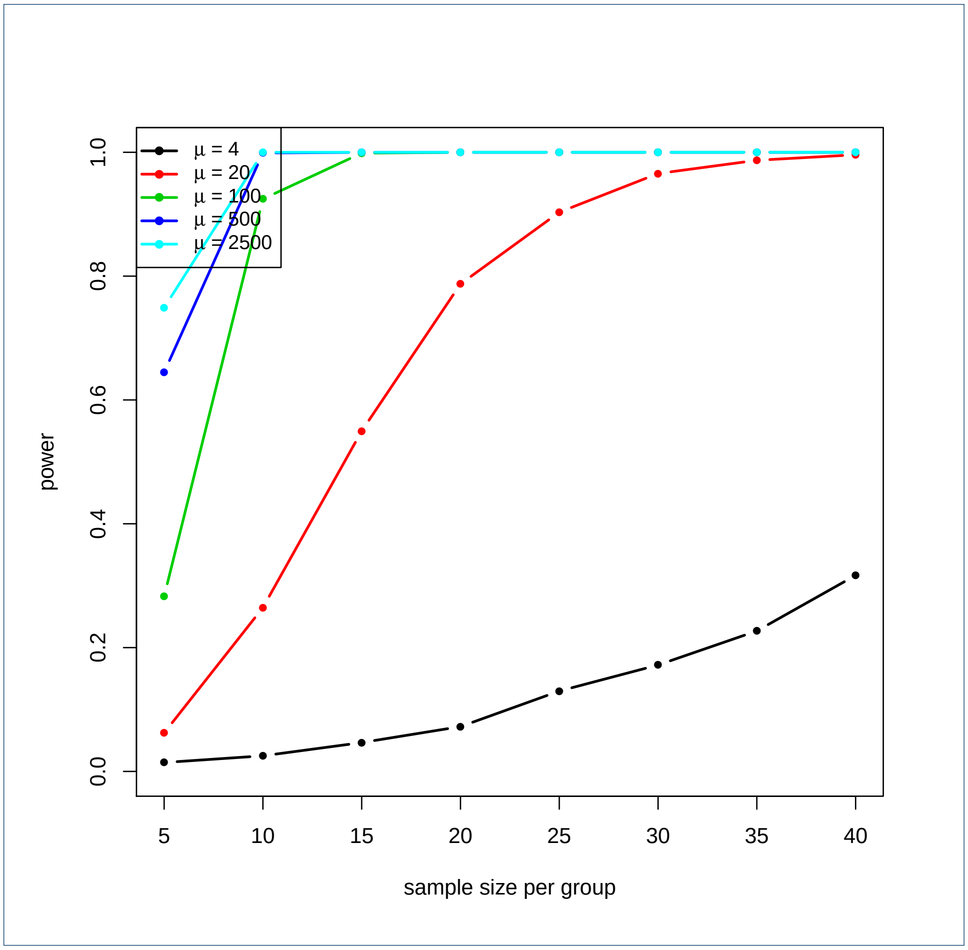

Figure S13: Power plot at $\gamma = 2$ for the Wald test with equal dispersion parameters assuming the high dependency functional form. Power was calculated at 8 different sample sizes and 5 different expression levels with $\gamma = 2$ under the alternative hypothesis and $\alpha = 0.001$. Power is higher for larger sample sizes and higher expression levels.

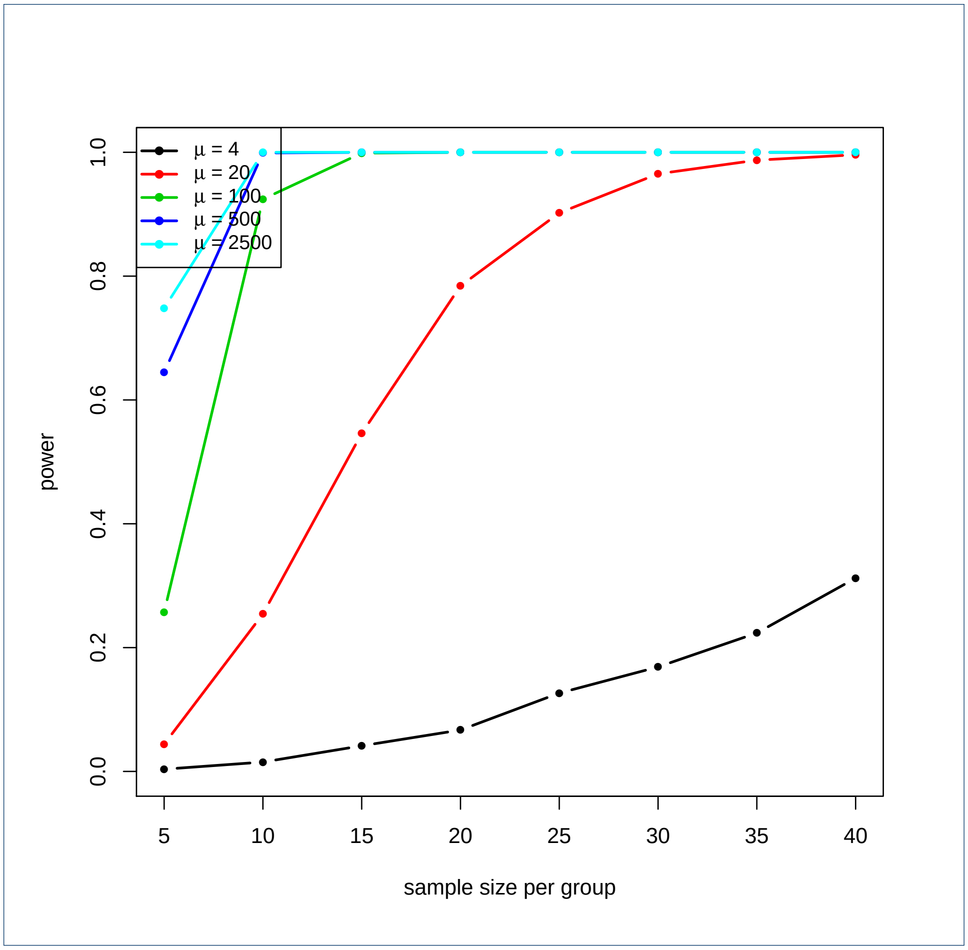

Figure S14: Power plot at $\gamma = 2$ for the LRT with equal dispersion parameters assuming the high dependency functional form. Power was calculated at 8 different sample sizes and 5 different expression levels with $\gamma = 2$ under the alternative hypothesis and $\alpha = 0.001$. Power is higher for larger sample sizes and higher expression levels.

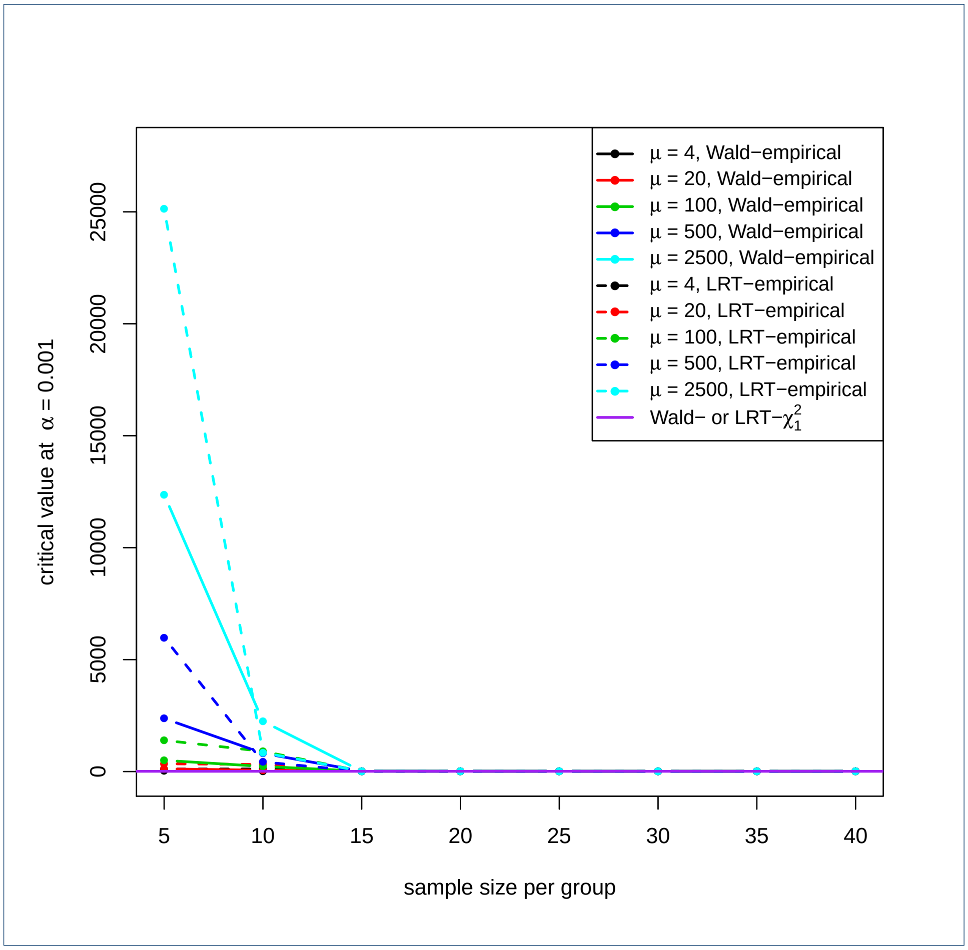

Figure S15: Critival values plot for both Wald test and LRT with equal dispersion parameters assuming the low dependency functional form. Critical values were calculated at the nominal false positive error rate of 0.001 from empirical percentile of null statisitics at 5 different mean expression levels for both Wald test (solid line) and LRT (dashed line), and for the chi-square distribution with 1 degree of freedom (purple line). Both Wald test and LRT with the empirical distribution have larger critical values than both Wald test and LRT with the chi-square distribution.

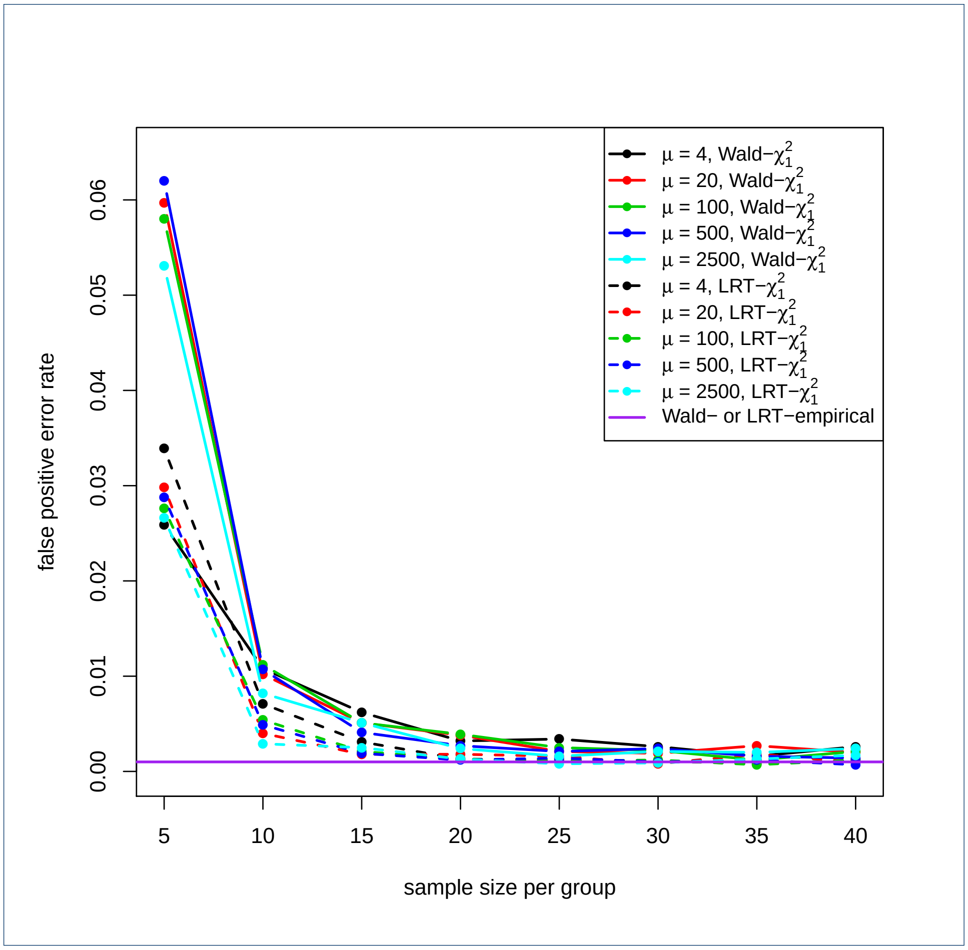

Figure S16: False positive error rate plot for both Wald test and LRT with equal dispersion parameters assuming the low dependency functional form. False positive error rate was calculated for both Wald test (solid line) and LRT (dashed line) following a chi-square distribution with 1 degree of freedom at 5 different mean expression levels. The nominal false positive error rate for both Wald and LRT with the empirical distribution is shown in purple line ($\alpha = 0.001$). Both tests with the chi-square distribution have the inflated false positive error rates.

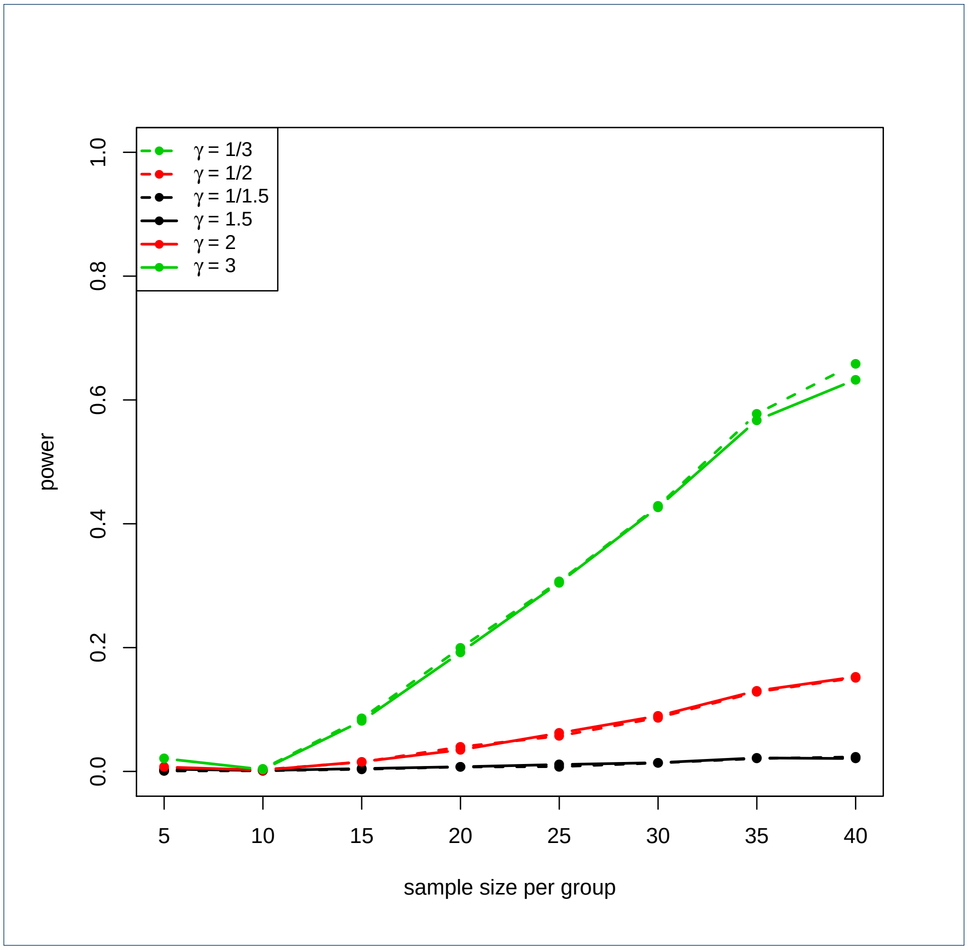

Figure S17: Power plot at $\mu = 100$ for the Wald test with equal dispersion parameters assuming the low dependency functional form. Power was calculated at 8 different sample sizes and 6 different fold changes under the alternative hypothesis with $\mu = 100$ and $\alpha = 0.001$. Power is higher for larger sample sizes and higher absolute fold changes.

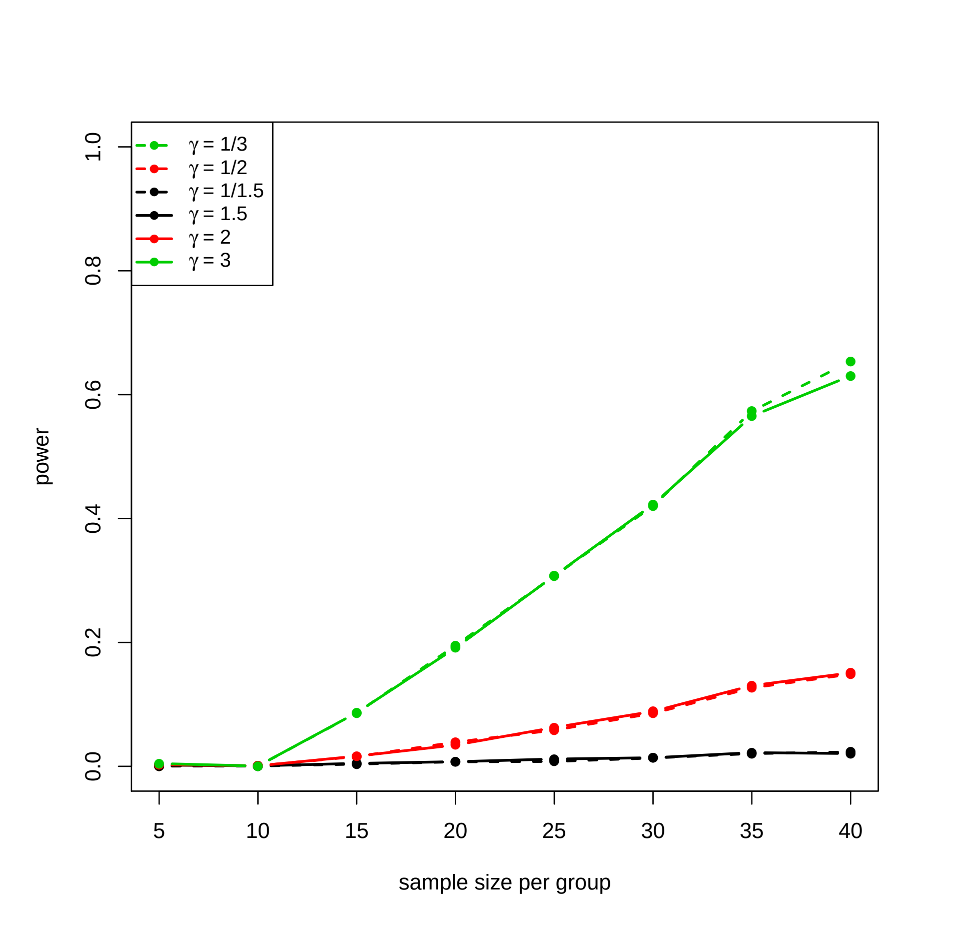

Figure S18: Power plot at $\mu = 100$ for the LRT with equal dispersion parameters assuming the low dependency functional form. Power was calculated at 8 different sample sizes and 6 different fold changes under the alternative hypothesis with $\mu = 100$ and $\alpha = 0.001$. Power is higher for larger sample sizes and higher absolute fold changes.

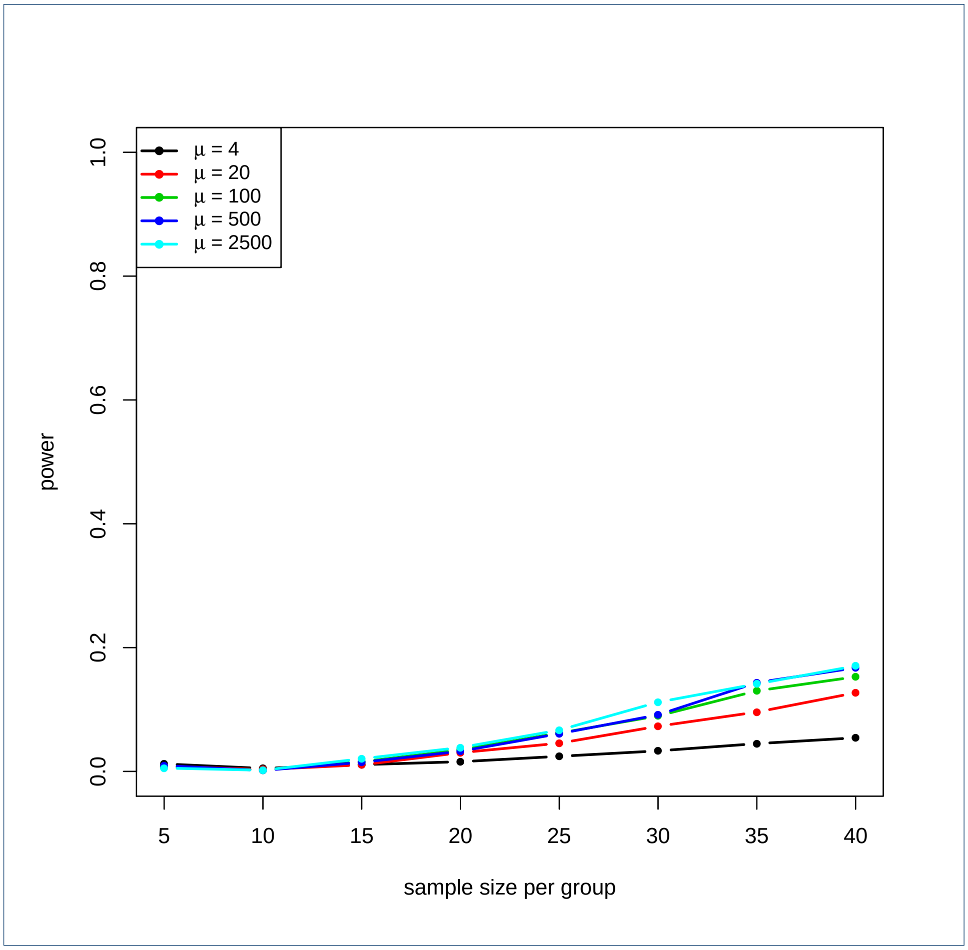

Figure S19: Power plot at $\gamma = 2$ for the Wald test with equal dispersion parameters assuming the low dependency functional form. Power was calculated at 8 different sample sizes and 5 different expression levels with $\gamma = 2$ under the alternative hypothesis and $\alpha = 0.001$. Power is higher for larger sample sizes and higher expression levels.

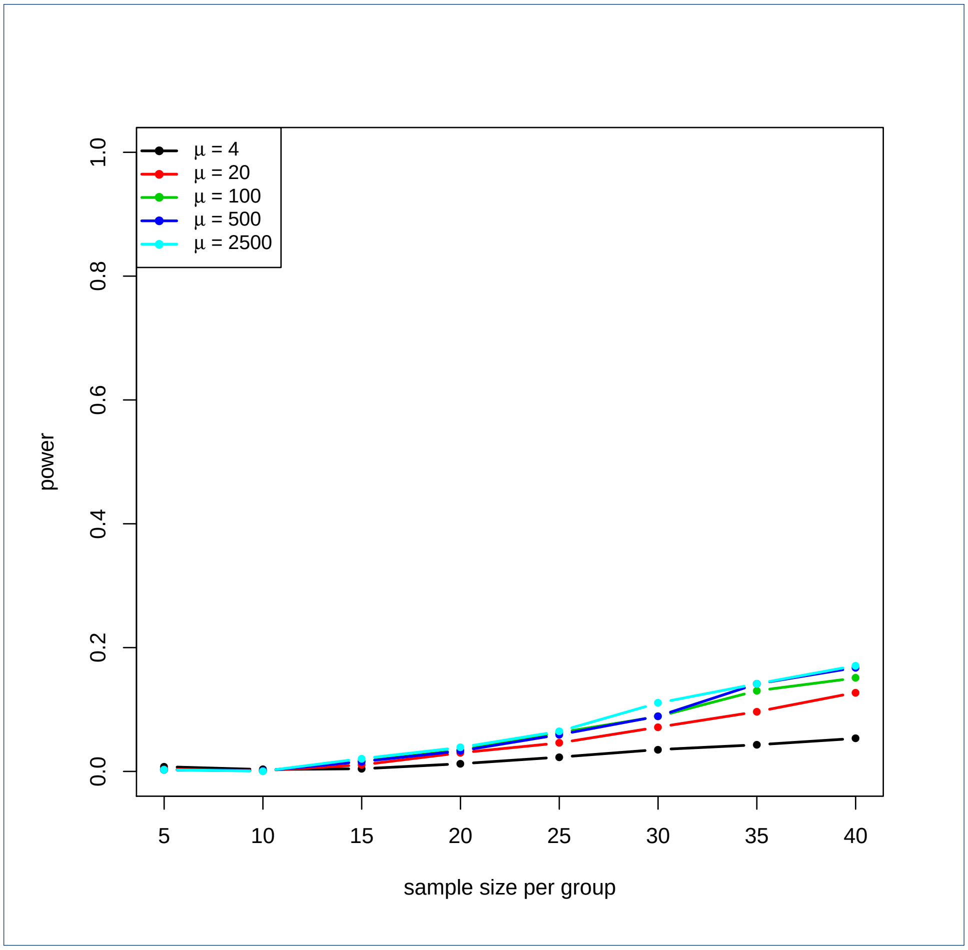

Figure S20: Power plot at $\gamma = 2$ for the LRT with equal dispersion parameters assuming the low dependency functional form. Power was calculated at 8 different sample sizes and 5 different expression levels with $\gamma = 2$ under the alternative hypothesis and $\alpha = 0.001$. Power is higher for larger sample sizes and higher expression levels.

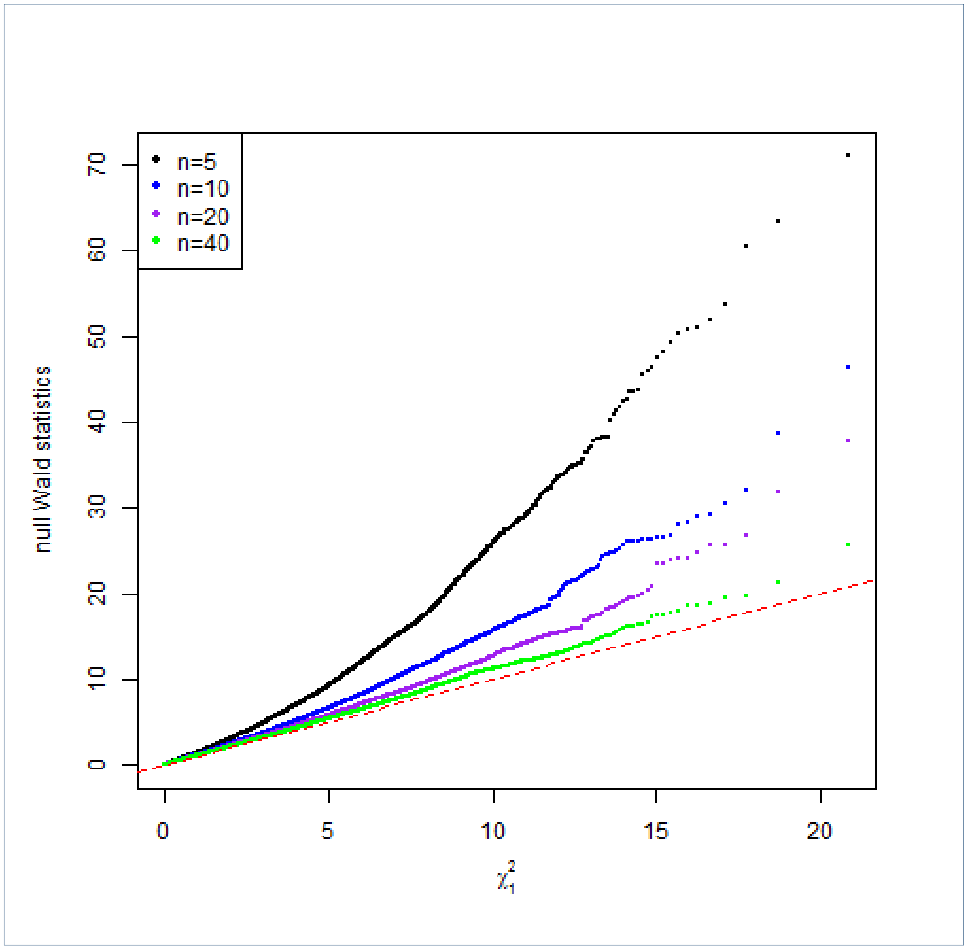

Figure S21: QQ plot of null Wald statistics with unequal dispersion parameters. Data were simulated 100,000 times with $\mu = 20$ under the null hypothesis. Sample sizes were set at $n = 5$,10,20,and 40. Wald test was used for testing mean difference between two conditions. The discrepancy of null Wald statistics from chi-square distribution with 1 degree of freedom is getting smaller with sample size increases.

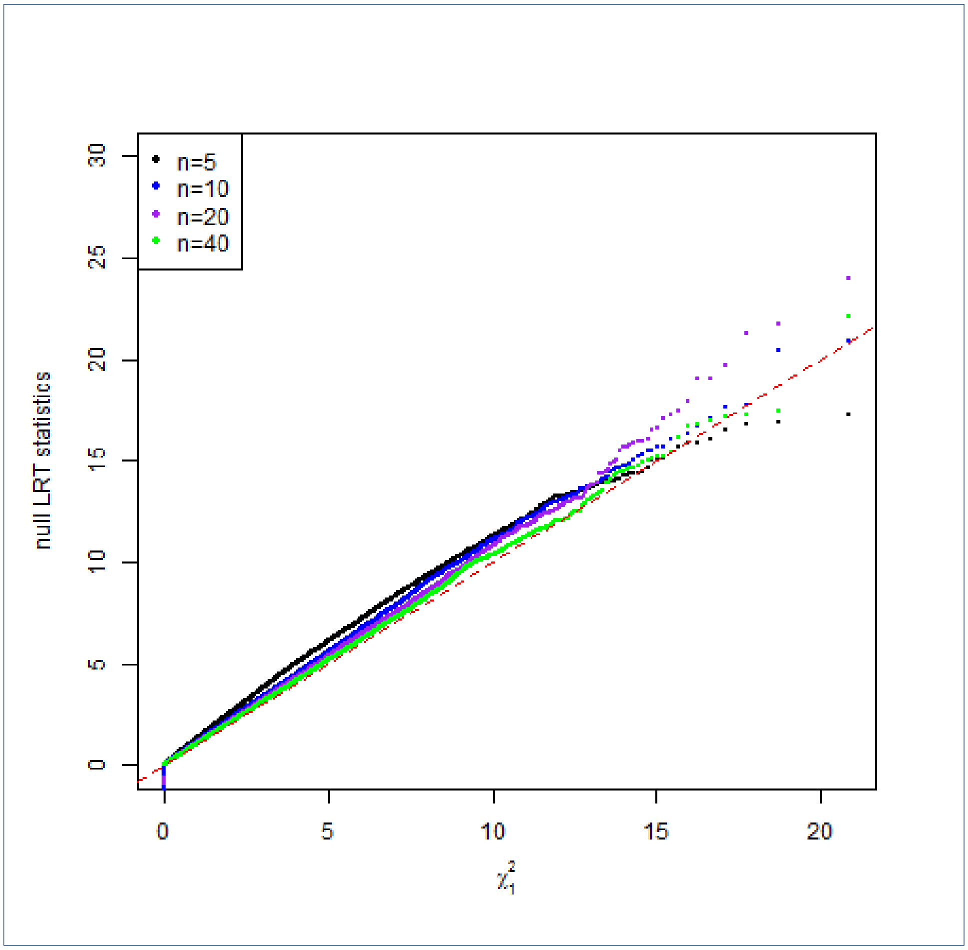

Figure S22: QQ plot of null LRT statistics with unequal dispersion parameters. Data were simulated 100, 000 times with $\mu = 20$ under the null hypothesis. Sample sizes were set at $n =$ 5,10,20,and 40. The LRT was used for testing mean difference between two conditions. The discrepancy of null LRT statistics from chi-square distribution with 1 degree of freedom is much smaller than the Wald test.

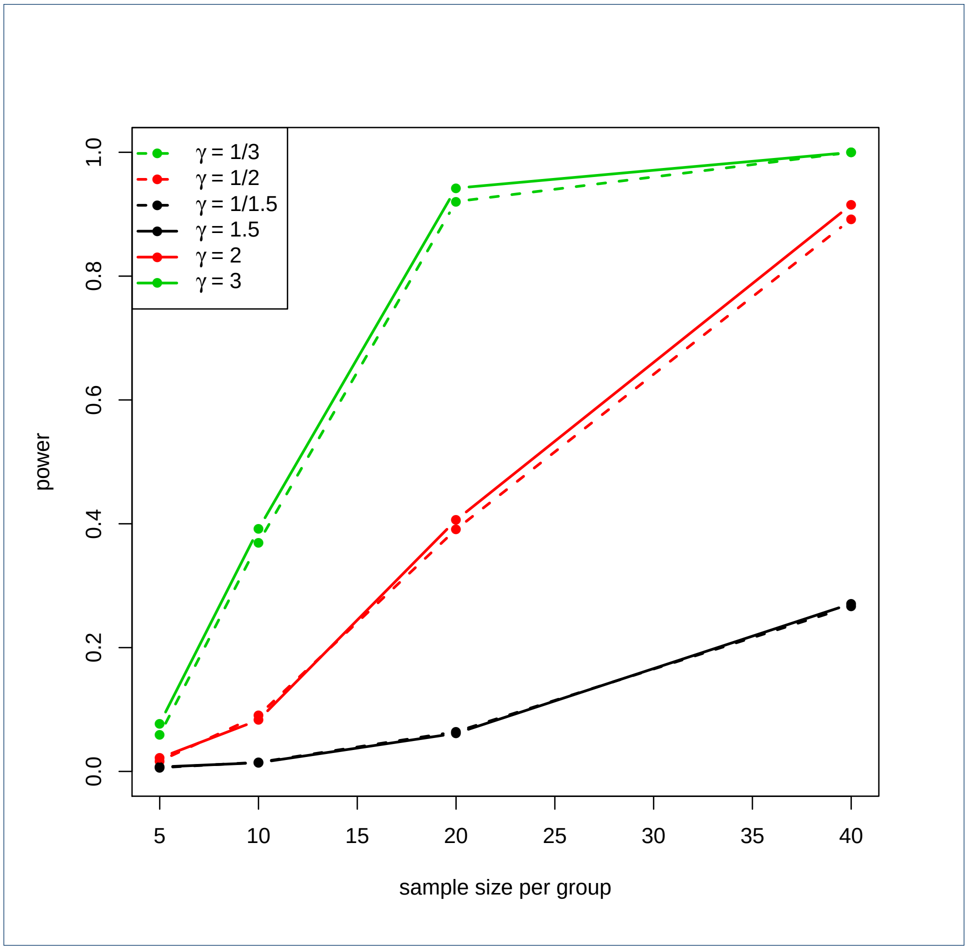

Figure S23: Power plot at $\mu = 20$ for the LRT with unequal dispersion parameters. Power was calculated at 4 different sample sizes and 6 different fold changes under the alternative hypothesis with $\mu = 20$ and $\alpha = 0.001$. Power is higher for larger sample sizes and higher absolute fold changes.

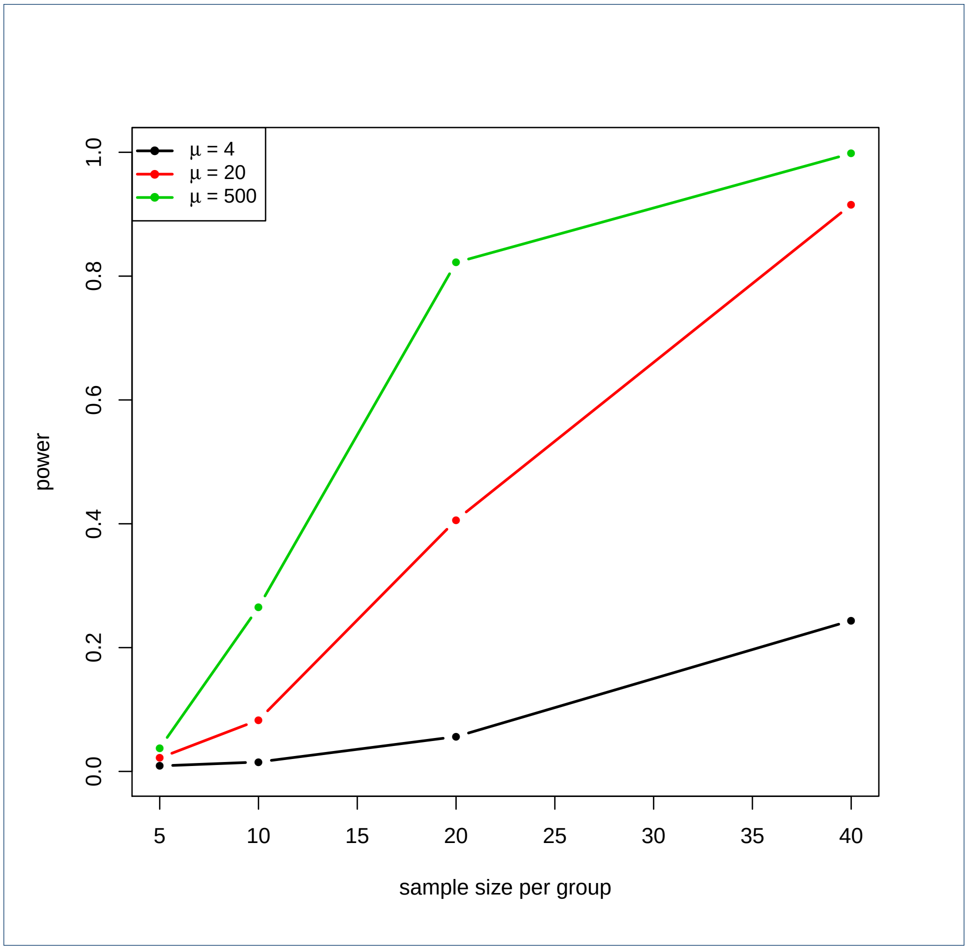

Figure S24: Power plot at $\gamma = 2$ for the LRT with unequal dispersion parameters. Power was calculated at 4 different sample sizes and 3 different expression levels with $\gamma = 2$ under the alternative hypothesis and $\alpha = 0.001$. Power is higher for larger sample sizes and higher expression levels.

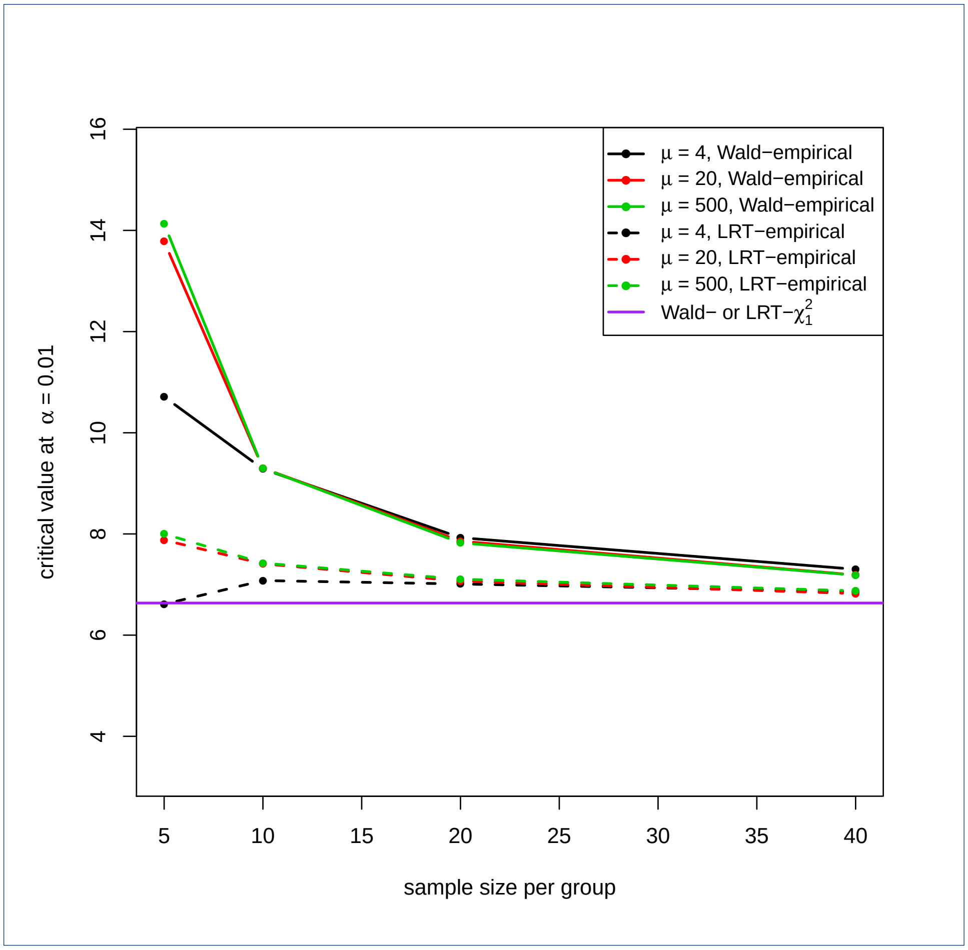

Figure S25: Critival values plot for both Wald test and LRT with unequal dispersion parameters at $\alpha = 0.01$. Critical values were calculated at the nominal false positive error rate of 0.01 from empirical percentile of null statisitics at 3 different mean expression levels for both Wald test (solid line) and LRT (dashed line), and for the chi-square distribution with 1 degree of freedom (purple line). Both Wald test and LRT with the empirical distribution have larger critical values than both Wald test and LRT with the chi-square distribution.

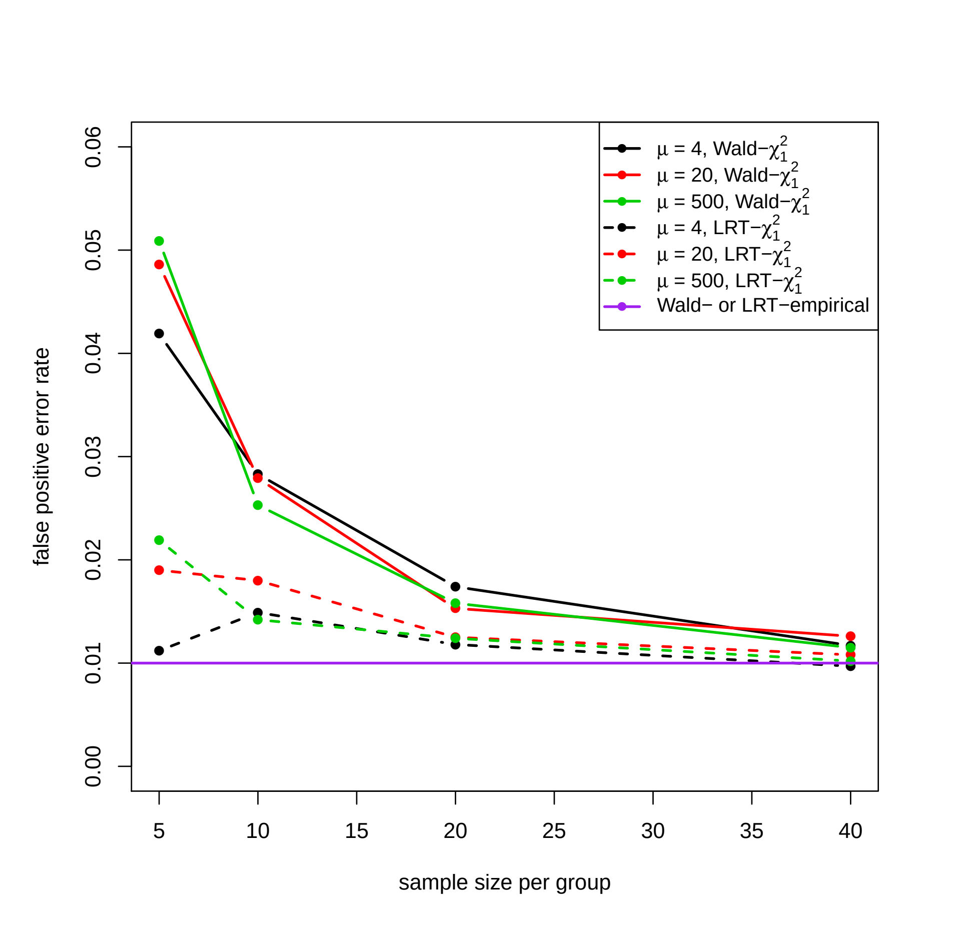

Figure S26: False positive error rate plot for both Wald test and LRT with unequal dispersion parameters at $\alpha = 0.01$. False positive error rate was calculated for both Wald test (solid line) and LRT (dashed line) following a chi-square distribution with 1 degree of freedom at 3 different mean expression levels. The nominal false positive error rate for both Wald and LRT with the empirical distribution is shown in purple line ($\alpha = 0.01$). Both tests with the chi-square distribution have the inflated false positive error rates.

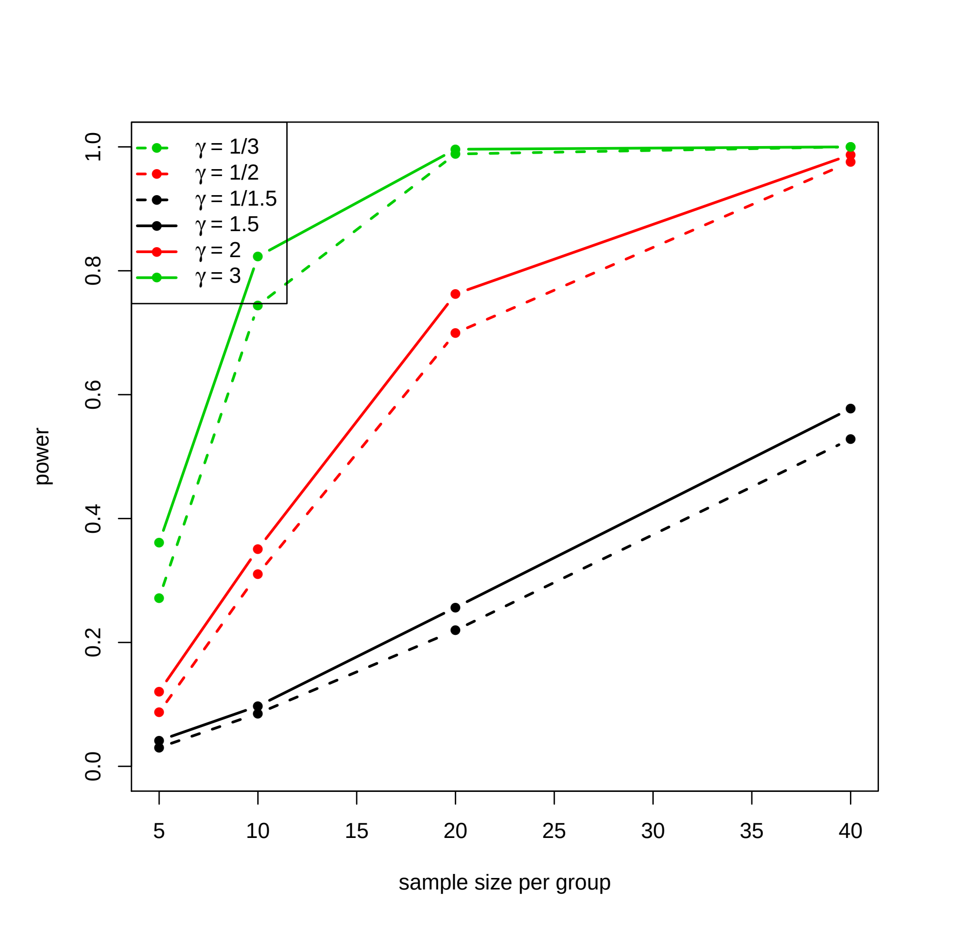

Figure S27: Power plot at $\mu = 20$ for the Wald test with unequal dispersion parameters at $\alpha = 0.01$. Power was calculated at 4 different sample sizes and 6 different fold changes under the alternative hypothesis with $\mu = 20$ and $\alpha = 0.01$. Power is higher for larger sample sizes and higher absolute fold changes.

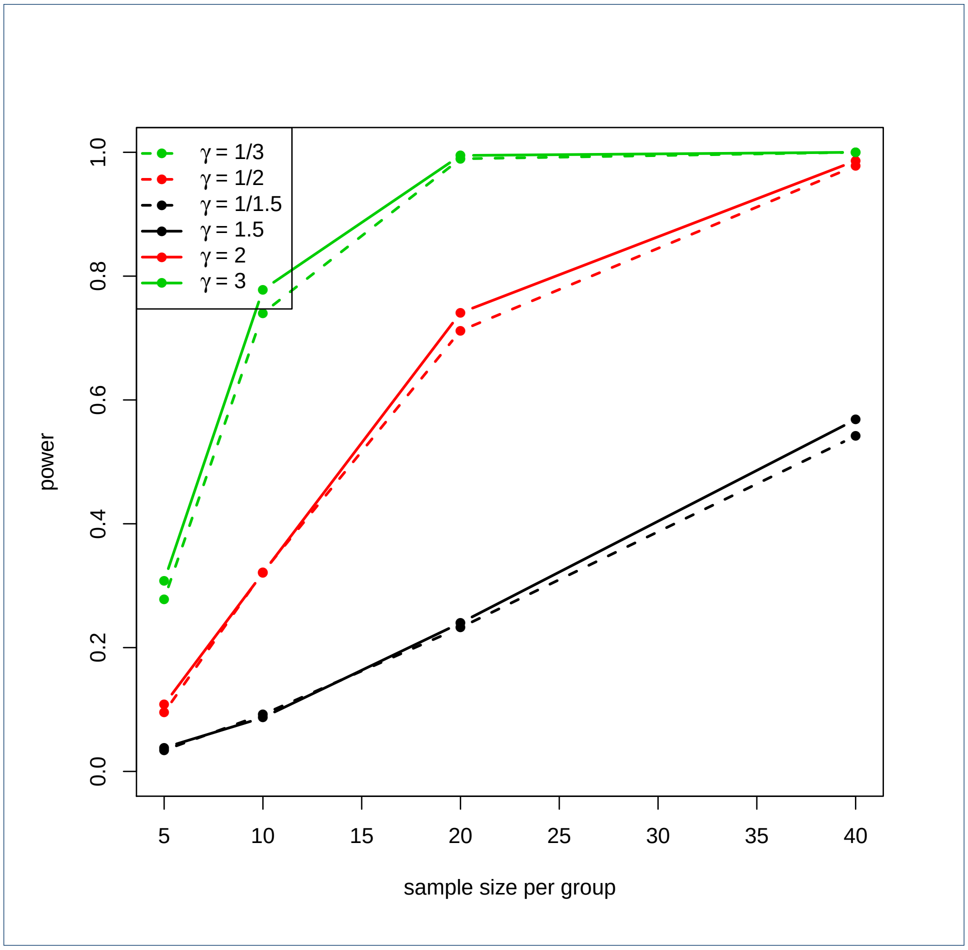

Figure S28: Power plot at $\mu = 20$ for the LRT with unequal dispersion parameters at $\alpha = 0.01$. Power was calculated at 4 different sample sizes and 6 different fold changes under the alternative hypothesis with $\mu = 20$ and $\alpha = 0.01$. Power is higher for larger sample sizes and higher absolute fold changes.

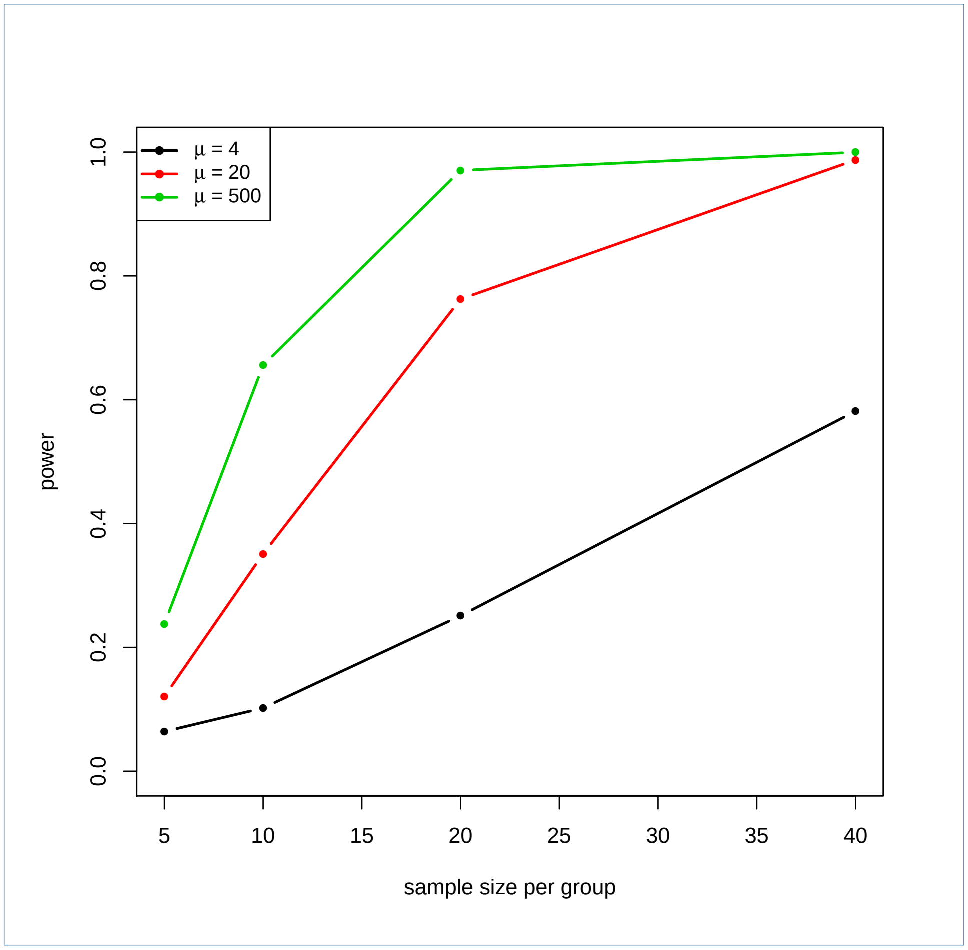

Figure S29: Power plot at $\gamma = 2$ for the Wald test with unequal dispersion parameters at $\alpha = 0.01$. Power was calculated at 4 different sample sizes and 3 different expression levels with $\gamma = 2$ under the alternative hypothesis and $\alpha = 0.01$. Power is higher for larger sample sizes and higher expression levels.

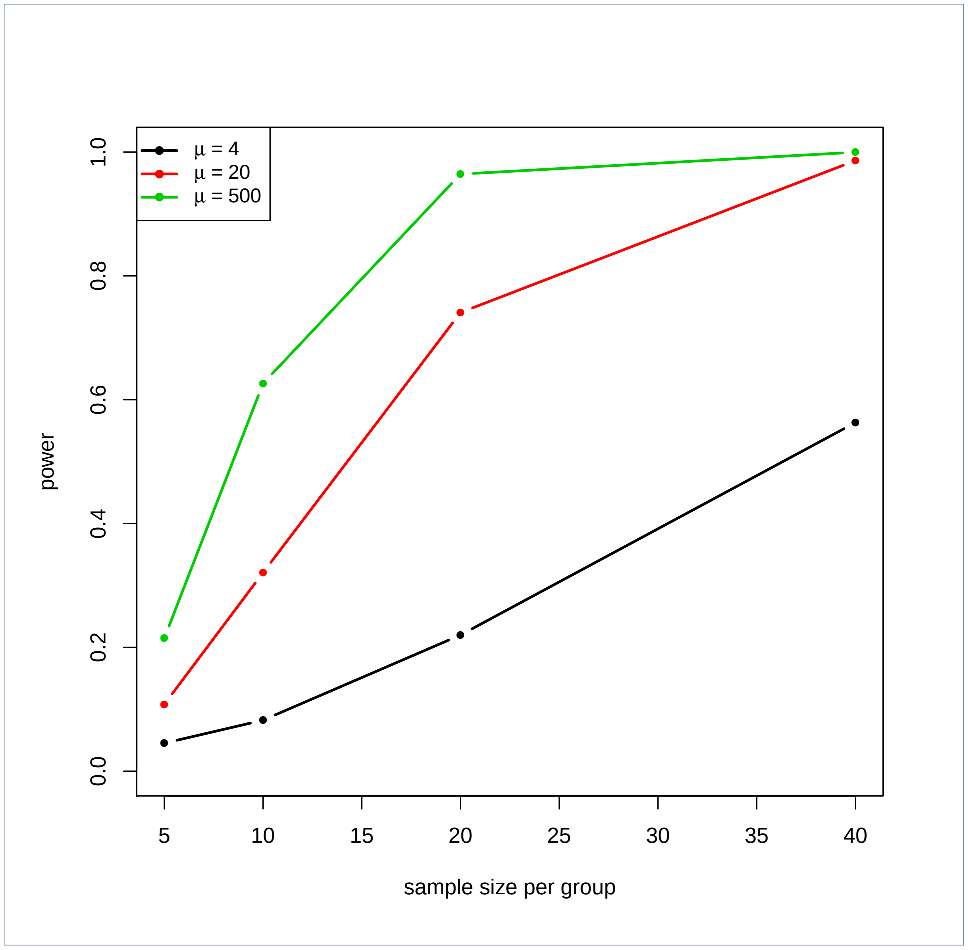

Figure S30: Power plot at $\gamma = 2$ for the LRT with unequal dispersion parameters at $\alpha = 0.01$. Power was calculated at 4 different sample sizes and 3 different expression levels with $\gamma = 2$ under the alternative hypothesis and $\alpha = 0.01$. Power is higher for larger sample sizes and higher expression levels.

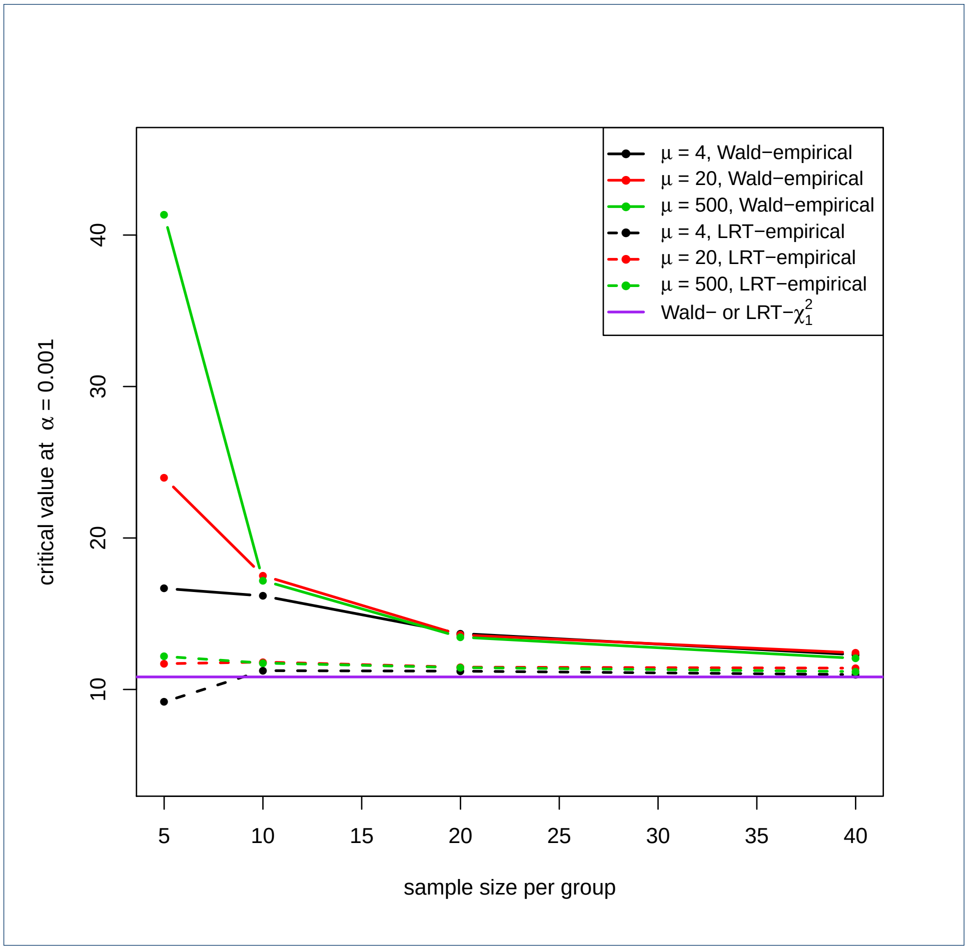

Figure S31: Critival values plot for both Wald test and LRT with unequal dispersion parameters assuming the high dependency functional form. Critical values were calculated at the nominal false positive error rate of 0.001 from empirical percentile of null statisitics at 3 different mean expression levels for both Wald test (solid line) and LRT (dashed line), and for the chi-square distribution with 1 degree of freedom (purple line). Both Wald test and LRT with the empirical distribution have larger critical values than both Wald test and LRT with the chi-square distribution.

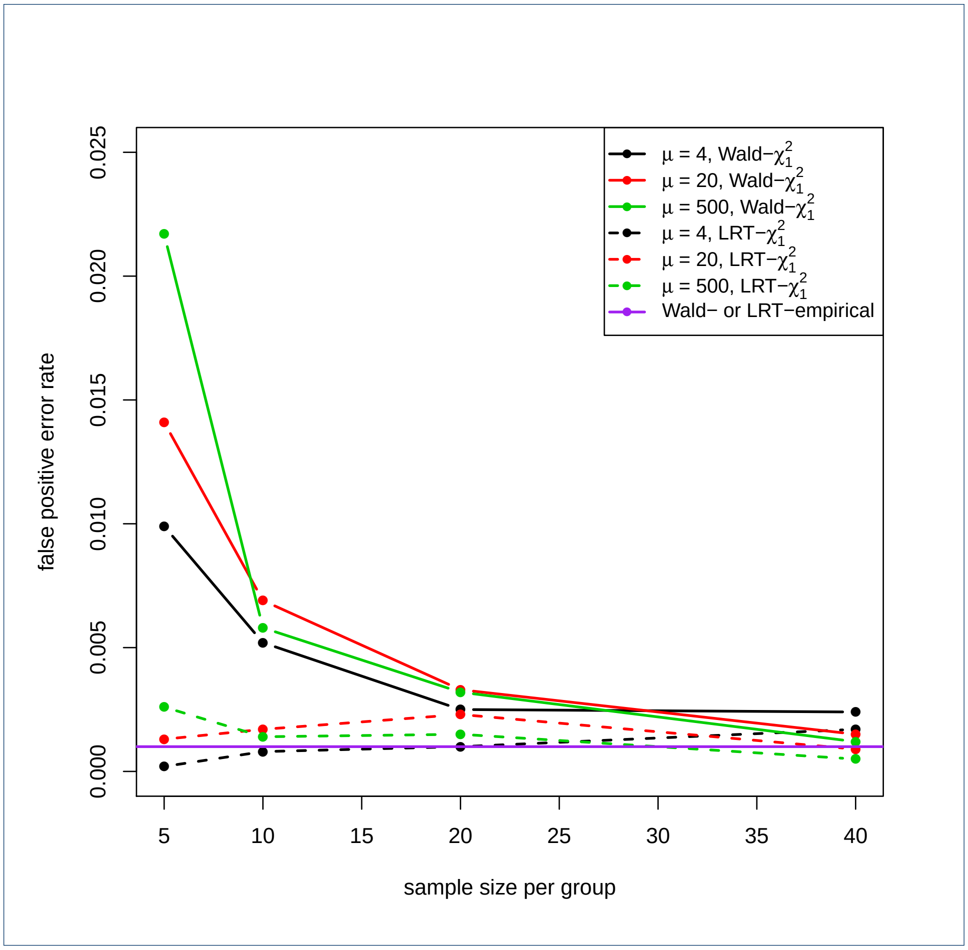

Figure S32: False positive error rate plot for both Wald test and LRT with unequal dispersion parameters assuming the high dependency functional form. False positive error rate was calculated for both Wald test (solid line) and LRT (dashed line) following a chi-square distribution with 1 degree of freedom at 3 different mean expression levels. The nominal false positive error rate for both Wald and LRT with the empirical distribution is shown in purple line ($\alpha = 0.001$). Both tests with the chi-square distribution have the inflated false positive error rates.

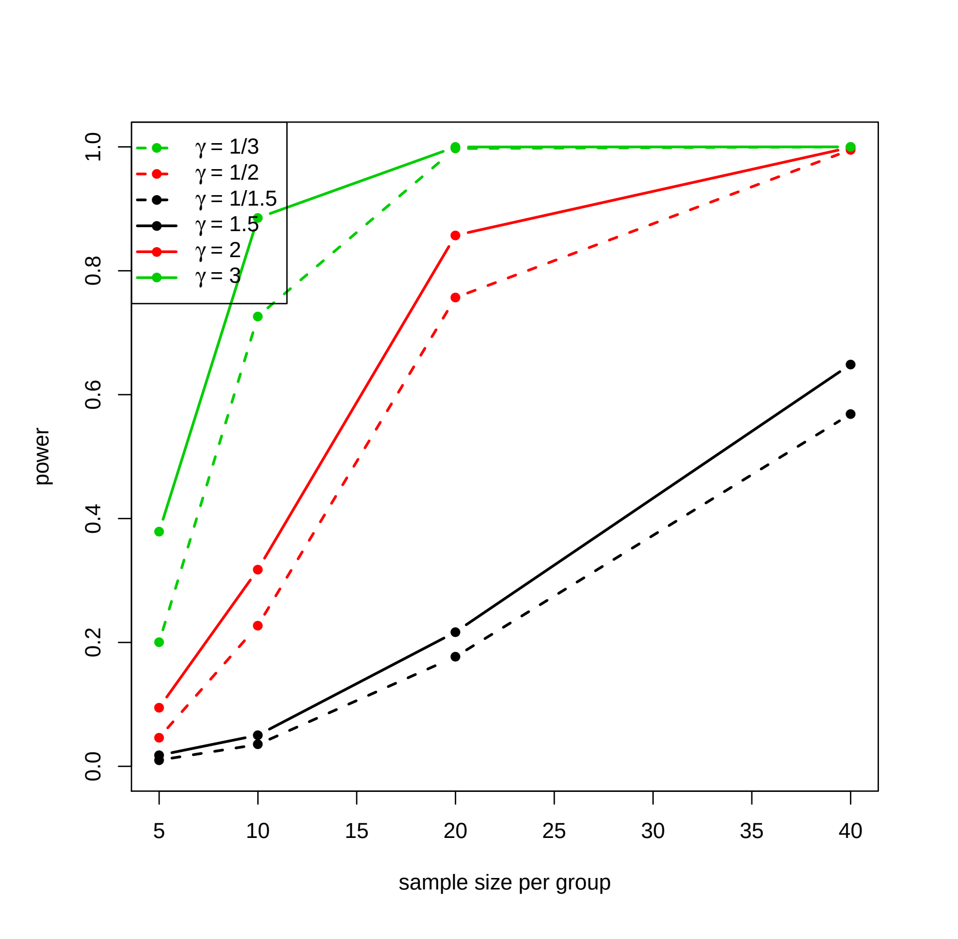

Figure S33: Power plot at $\mu = 20$ for the Wald test with unequal dispersion parameters assuming the high dependency functional form. Power was calculated at 4 different sample sizes and 6 different fold changes under the alternative hypothesis with $\mu = 20$ and $\alpha = 0.001$. Power is higher for larger sample sizes and higher absolute fold changes.

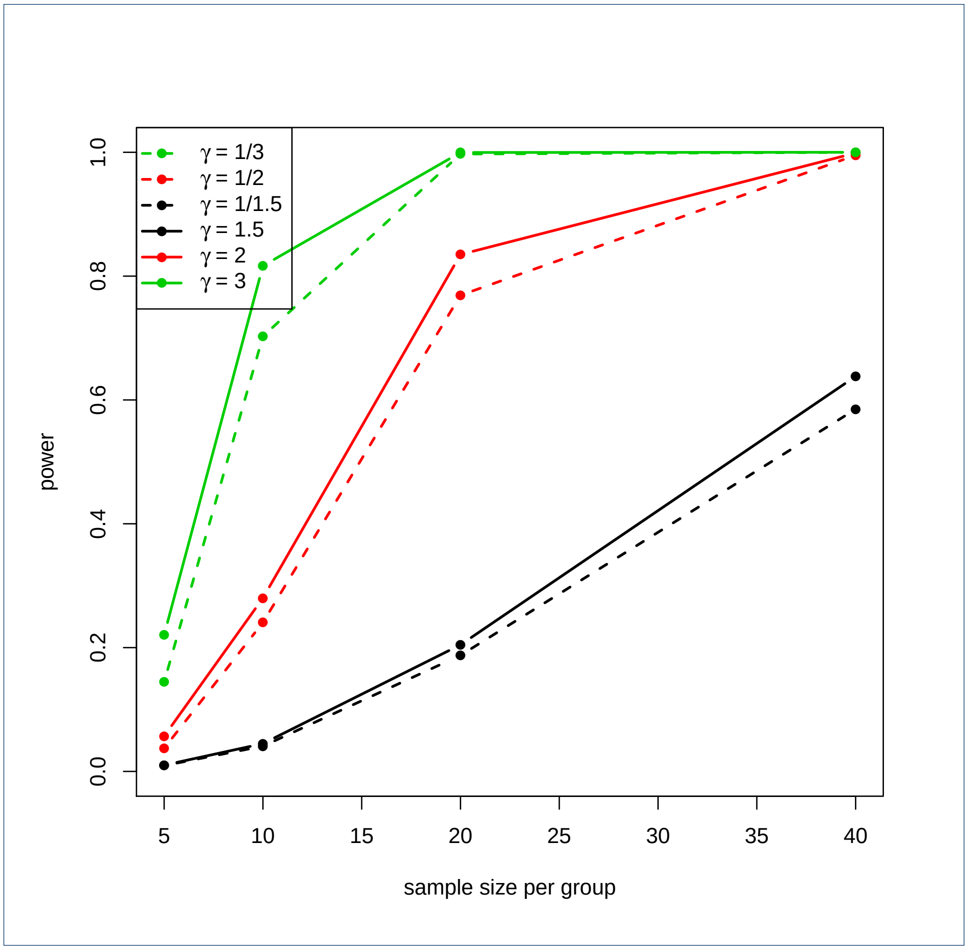

Figure S34: Power plot at $\mu = 20$ for the LRT with unequal dispersion parameters assuming the high dependency functional form. Power was calculated at 4 different sample sizes and 6 different fold changes under the alternative hypothesis with $\mu = 20$ and $\alpha = 0.001$. Power is higher for larger sample sizes and higher absolute fold changes.

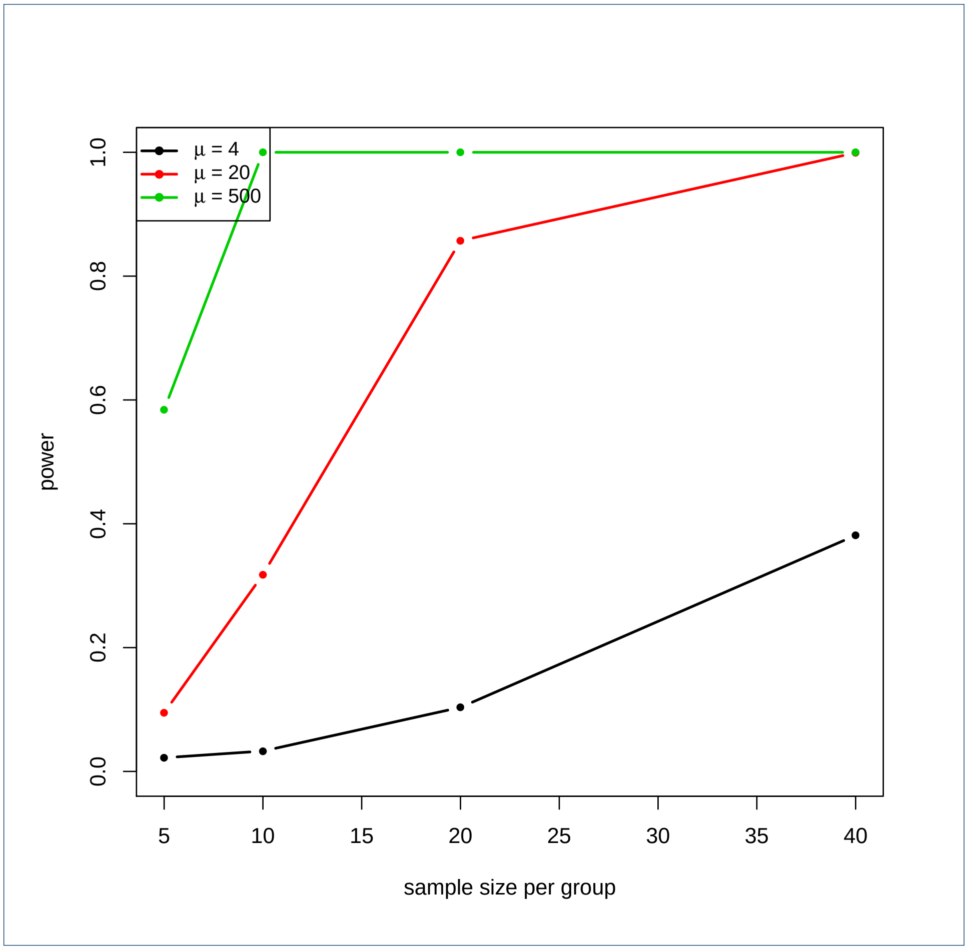

Figure S35: Power plot at $\gamma = 2$ for the Wald test with unequal dispersion parameters assuming the high dependency functional form. Power was calculated at 4 different sample sizes and 3 different expression levels with $\gamma = 2$ under the alternative hypothesis and $\alpha = 0.001$. Power is higher for larger sample sizes and higher expression levels.

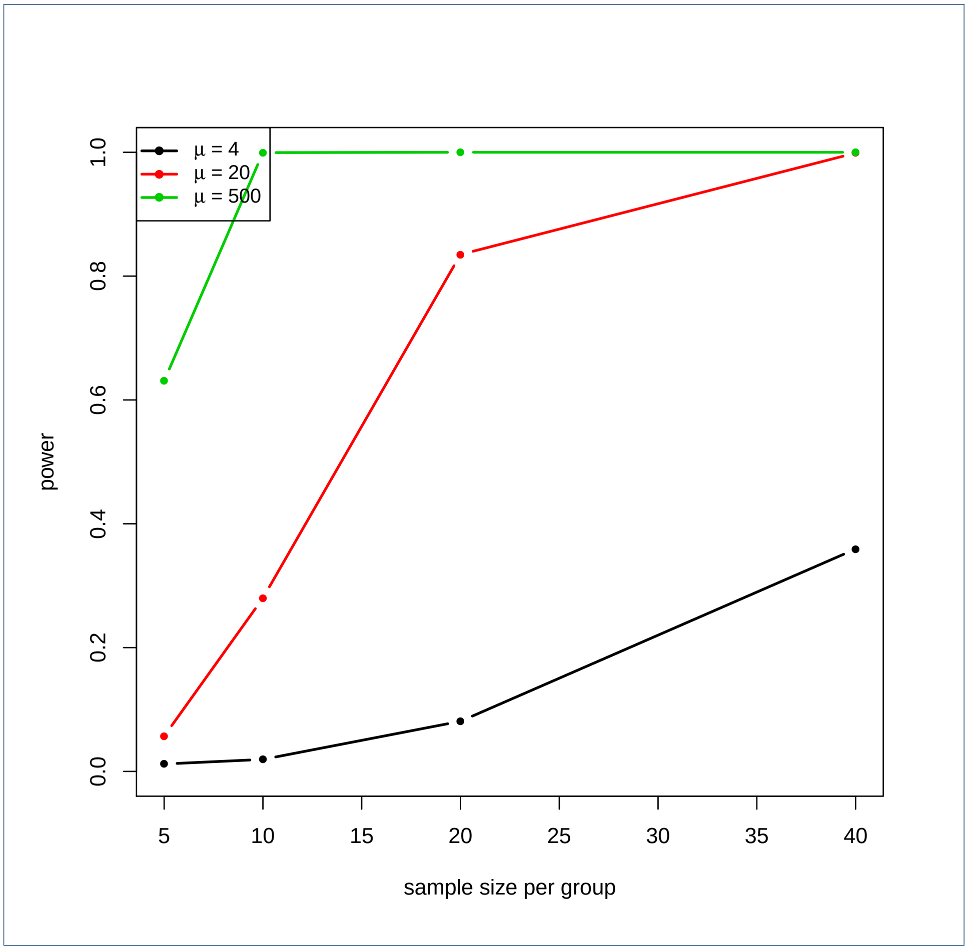

Figure S36: Power plot at $\gamma = 2$ for the LRT with unequal dispersion parameters assuming the high dependency functional form. Power was calculated at 4 different sample sizes and 3 different expression levels with $\gamma = 2$ under the alternative hypothesis and $\alpha = 0.001$. Power is higher for larger sample sizes and higher expression levels.

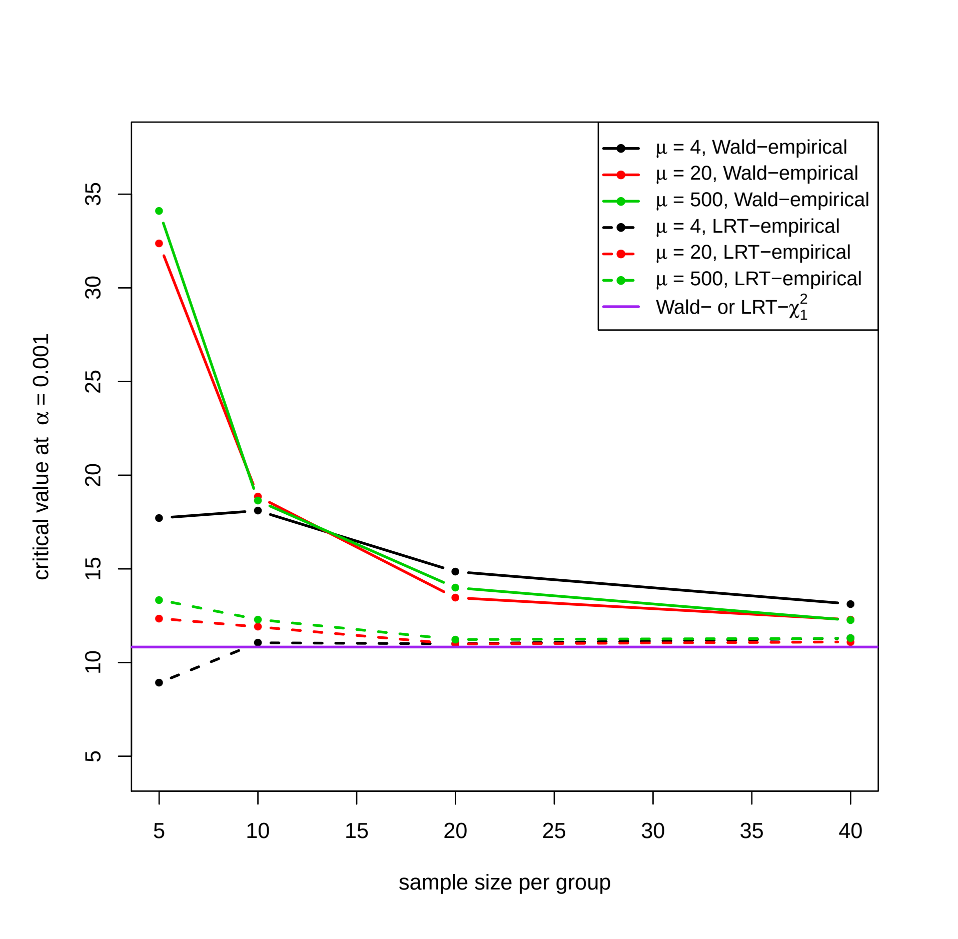

Figure S37: Critival values plot for both Wald test and LRT with unequal dispersion parameters assuming the low dependency functional form. Critical values were calculated at the nominal false positive error rate of 0.001 from empirical percentile of null statisitics at 3 different mean expression levels for both Wald test (solid line) and LRT (dashed line), and for the chi-square distribution with 1 degree of freedom (purple line). Both Wald test and LRT with the empirical distribution have larger critical values than both Wald test and LRT with the chi-square distribution.

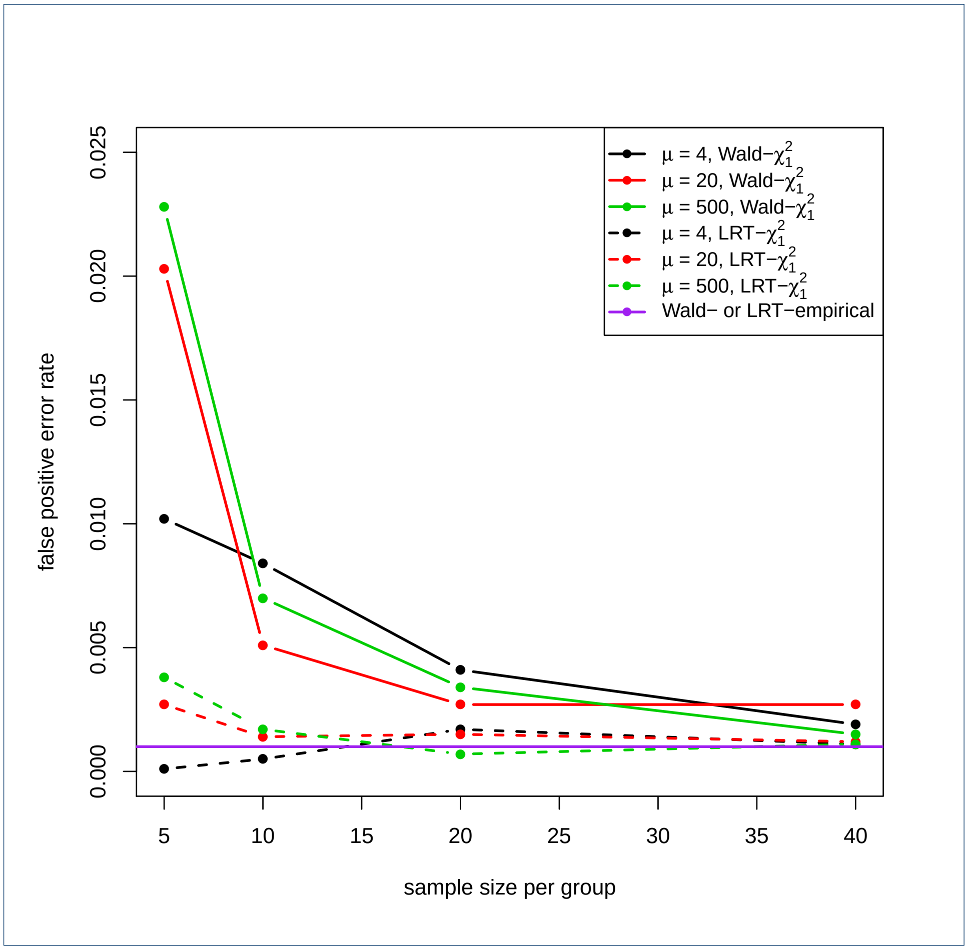

Figure S38: False positive error rate plot for both Wald test and LRT with unequal dispersion parameters assuming the low dependency functional form. False positive error rate was calculated for both Wald test (solid line) and LRT (dashed line) following a chi-square distribution with 1 degree of freedom at 3 different mean expression levels. The nominal false positive error rate for both Wald and LRT with the empirical distribution is shown in purple line ($\alpha = 0.001$). Both tests with the chi-square distribution have the inflated false positive error rates.

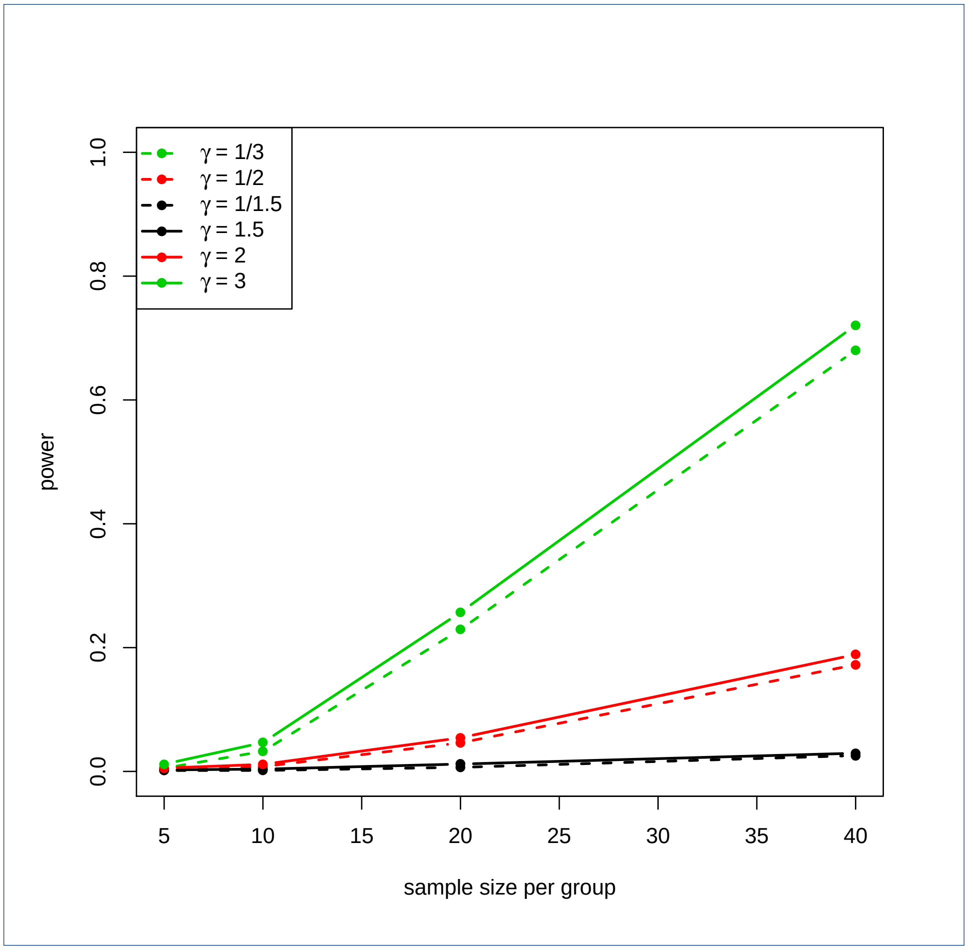

Figure S39: Power plot at $\mu = 20$ for the Wald test with unequal dispersion parameters assuming the low dependency functional form. Power was calculated at 4 different sample sizes and 6 different fold changes under the alternative hypothesis with $\mu = 20$ and $\alpha = 0.001$. Power is higher for larger sample sizes and higher absolute fold changes.

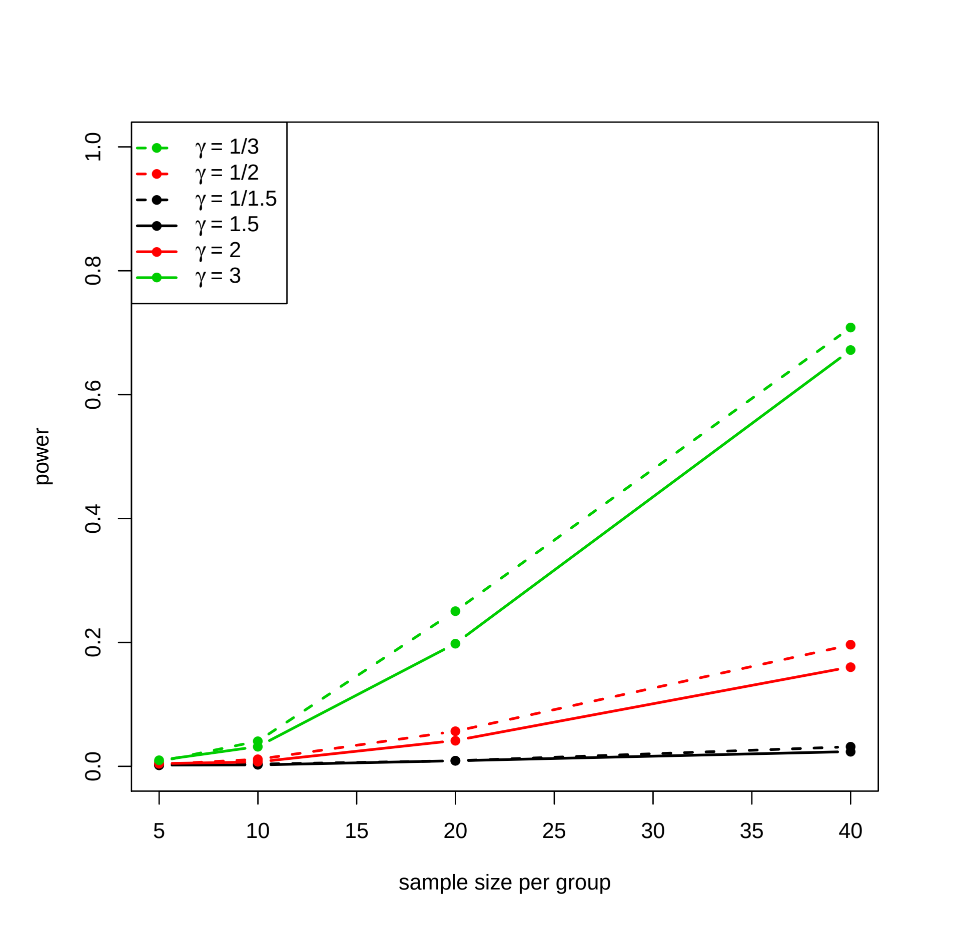

Figure S40: Power plot at $\mu = 20$ for the LRT with unequal dispersion parameters assuming the low dependency functional form. Power was calculated at 4 different sample sizes and 6 different fold changes under the alternative hypothesis with $\mu = 20$ and $\alpha = 0.001$. Power is higher for larger sample sizes and higher absolute fold changes.

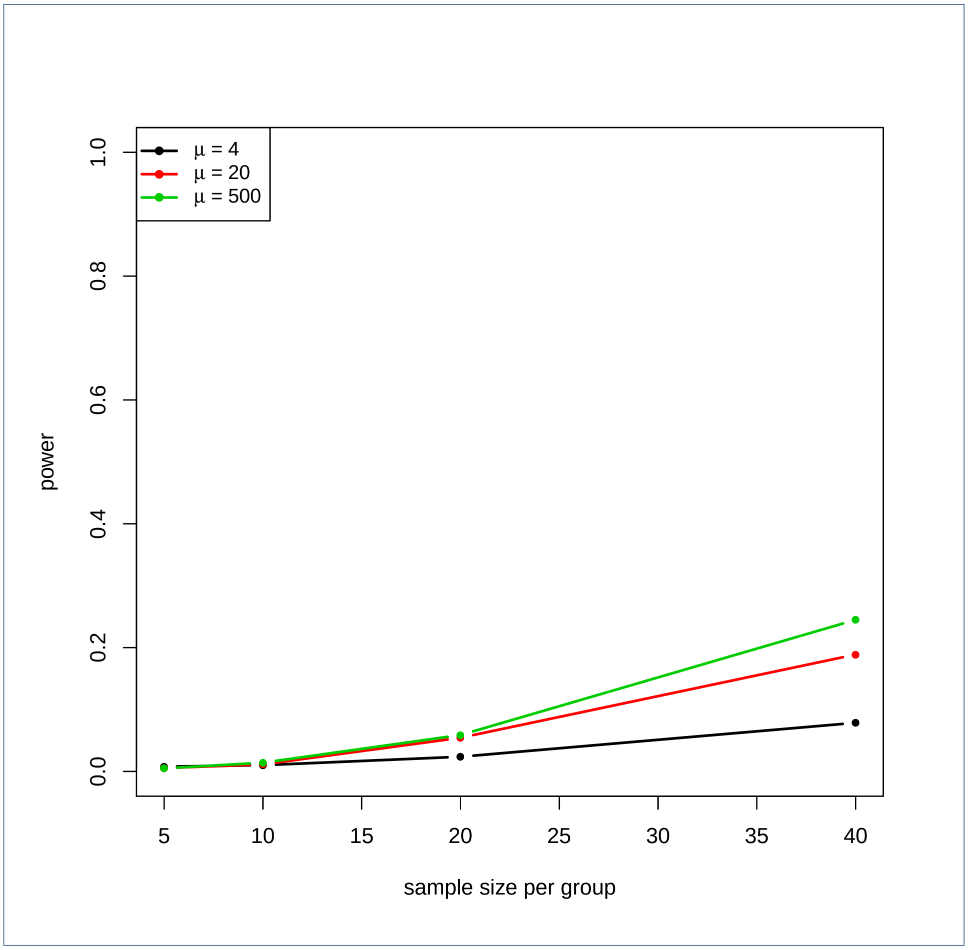

Figure S41: Power plot at $\gamma = 2$ for the Wald test with unequal dispersion parameters assuming the low dependency functional form. Power was calculated at 4 different sample sizes and 3 different expression levels with $\gamma = 2$ under the alternative hypothesis and $\alpha = 0.001$. Power is higher for larger sample sizes and higher expression levels.

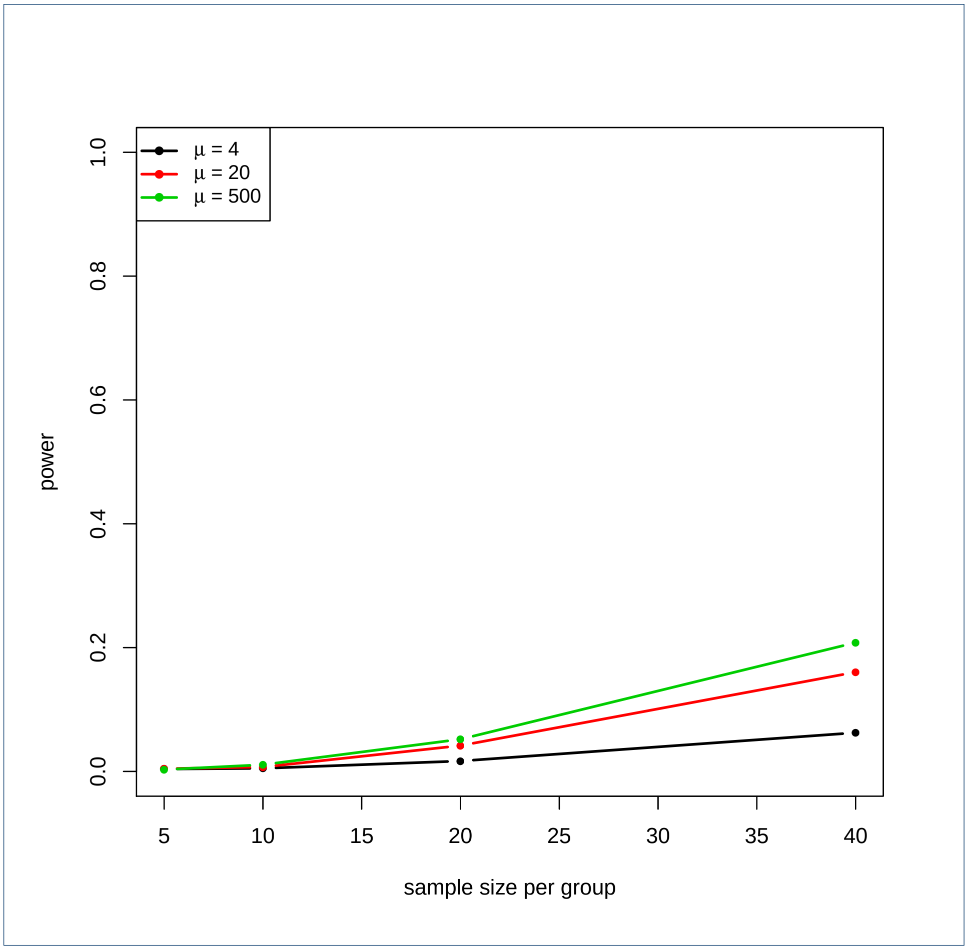

Figure S42: Power plot at $\gamma = 2$ for the LRT with unequal dispersion parameters assuming the low dependency functional form. Power was calculated at 4 different sample sizes and 3 different expression levels with $\gamma = 2$ under the alternative hypothesis and $\alpha = 0.001$. Power is higher for larger sample sizes and higher expression levels.

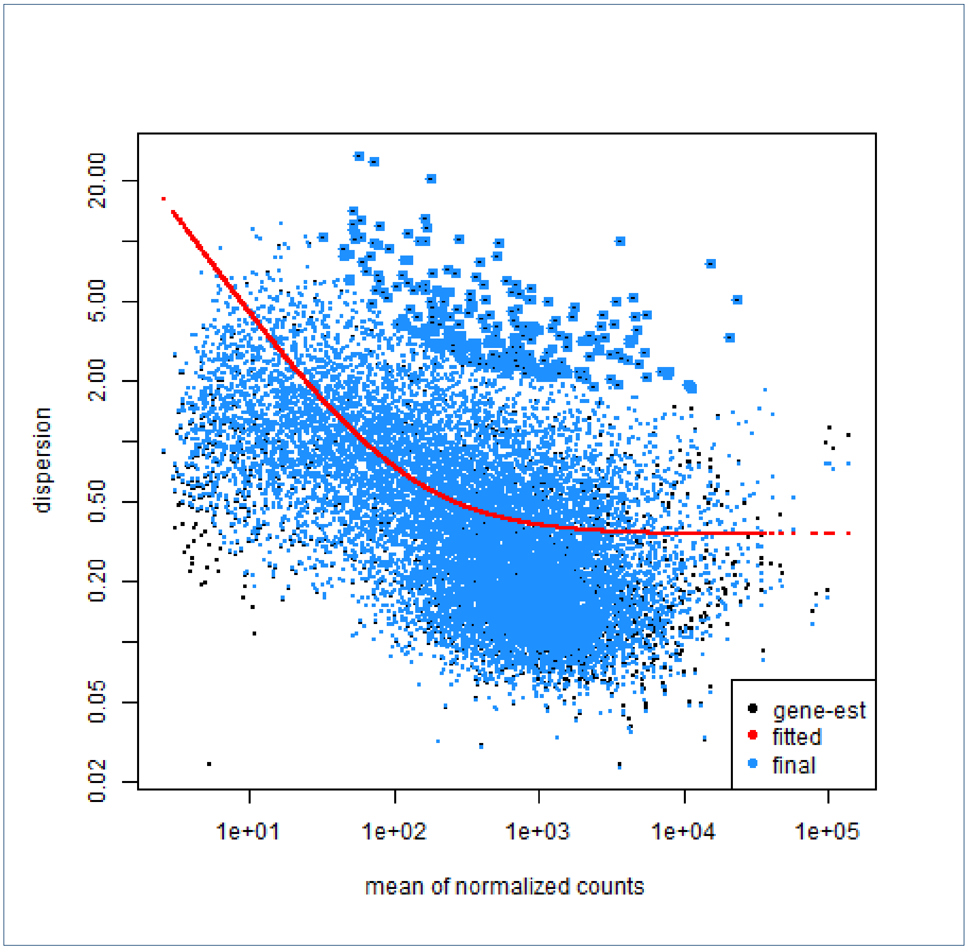

Figure S43: Mean-dispersion functional form of TCGA breast cancer data set. *DESeq2* method was applied on the TCGA breast cancer RNA-Seq data set. The plot shows the estimated mean-dispersion function form (red dots) relative to the mean of normalized counts.

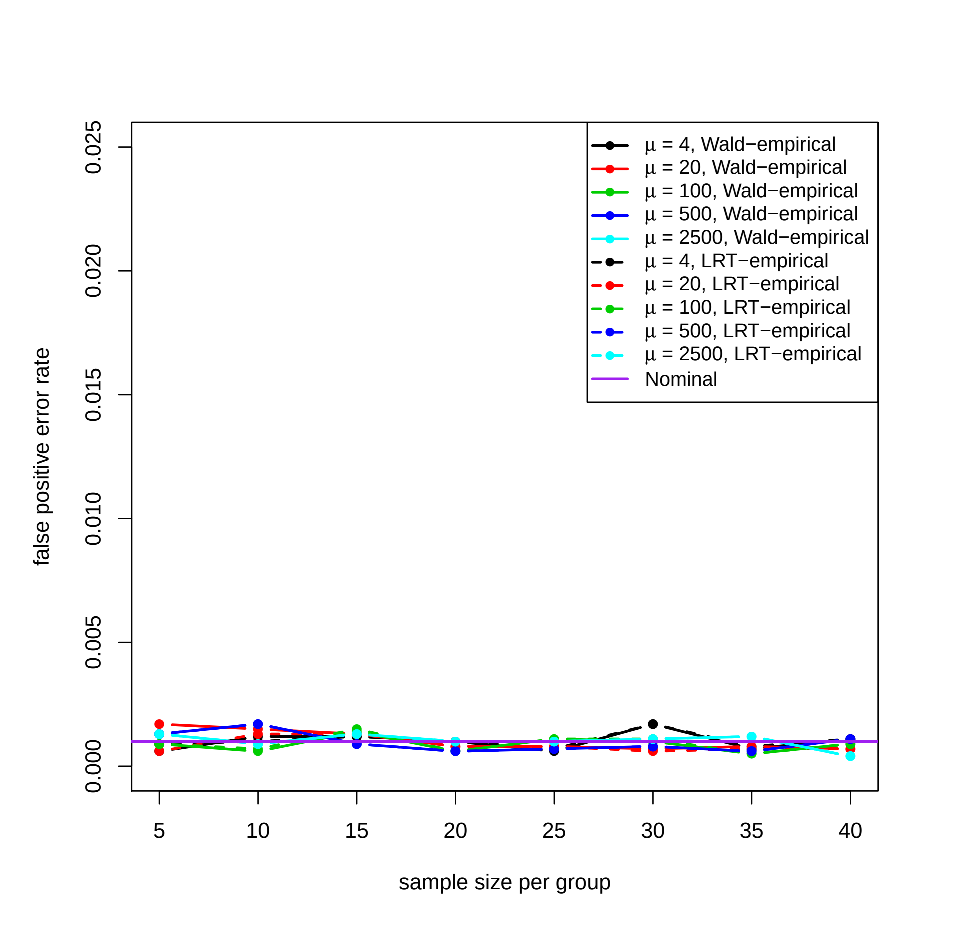

Figure S44: False positive error rate plot for both Wald test and LRT with equal dispersion parameters. False positive error rate was calculated for both Wald test (solid line) and LRT (dashed line) at 5 different mean expression levels using the critical values from the empirical distribution of a different simulation. The nominal false positive error rate is shown in purple line ($\alpha = 0.001$).

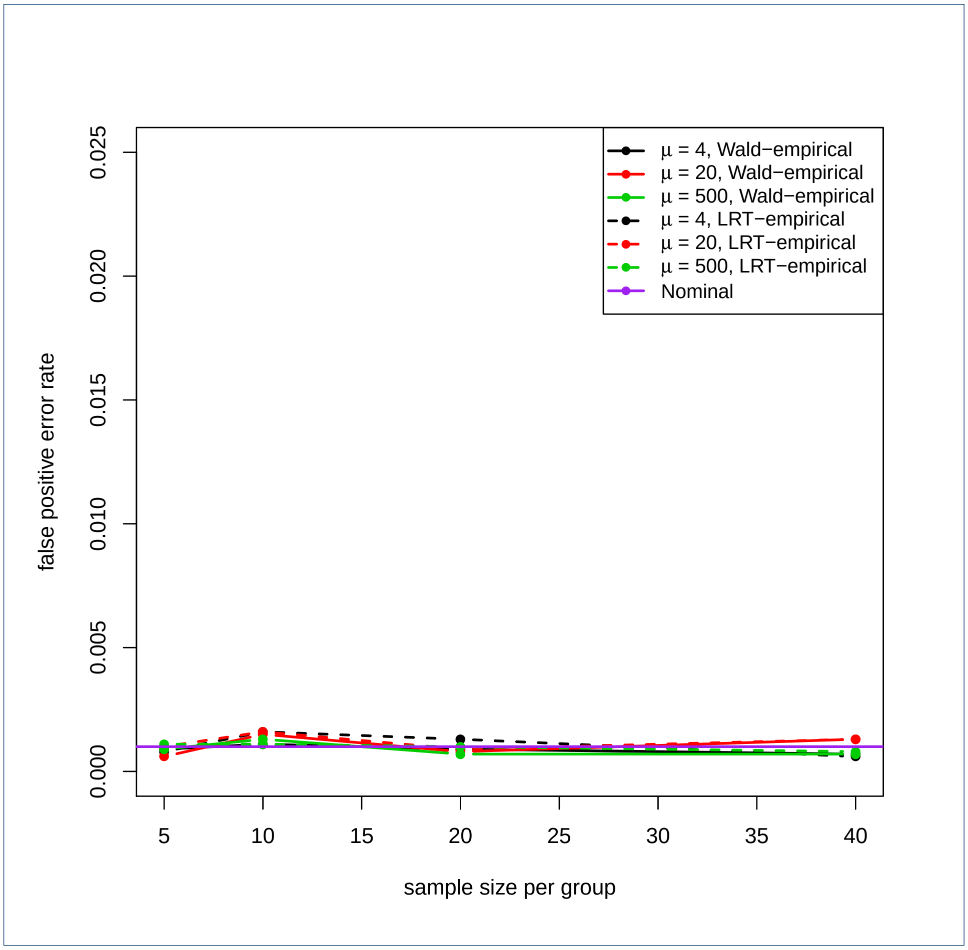

Figure S45**: False positive error rate plot for both Wald test and LRT with unequal dispersion parameters.** False positive error rate was calculated for both Wald test (solid line) and LRT (dashed line) at 5 different mean expression levels using the critical values from the empirical distribution of a different simulation. The nominal false positive error rate is shown in purple line ($\alpha = 0.001$).
